# Fat-to-blood recirculation of partially dysfunctional PD-1^+^CD4 Tconv cells is associated with dysglycemia in human obesity

**DOI:** 10.1016/j.isci.2024.109032

**Published:** 2024-02-01

**Authors:** Anna Giovenzana, Eugenia Bezzecchi, Anita Bichisecchi, Sara Cardellini, Francesca Ragogna, Federica Pedica, Federica Invernizzi, Luigi Di Filippo, Valentina Tomajer, Francesca Aleotti, Giulia M. Scotti, Carlo Socci, Giovanni Cesana, Stefano Olmi, Marco J. Morelli, Massimo Falconi, Andrea Giustina, Chiara Bonini, Lorenzo Piemonti, Eliana Ruggiero, Alessandra Petrelli

**Affiliations:** 1 IRCCS Ospedale San Raffaele, Milan, Italy; 2 San Marco Hospital GSD, Zingonia, Bergamo, Italy; 3 Università Vita-Salute San Raffaele, Milan, Italy

**Keywords:** Health sciences, Obesity medicine

## Abstract

Obesity is characterized by the accumulation of T cells in insulin-sensitive tissues, including the visceral adipose tissue (VAT), that can interfere with the insulin signaling pathway eventually leading to insulin resistance (IR) and type 2 diabetes. Here, we found that PD-1^+^CD4 conventional T (Tconv) cells, endowed with a transcriptomic and functional profile of partially dysfunctional cells, are diminished in VAT of obese patients with dysglycemia (OB-Dys), without a concomitant increase in apoptosis. These cells showed enhanced capacity to recirculate into the bloodstream and had a non-restricted TCRβ repertoire divergent from that of normoglycemic obese and lean individuals. PD-1^+^CD4 Tconv were reduced in the circulation of OB-Dys, exhibited an altered migration potential, and were detected in the liver of patients with non-alcoholic steatohepatitis. The findings suggest a potential role for partially dysfunctional PD-1^+^CD4 Tconv cells as inter-organ mediators of IR in obese patients with dysglycemic.

## Highlights

• Dysglycemic obesity associates with reduced partially dysfunctional PD-1+CD4 Tconv in VAT
• Dysglycemia induces alterations in the TCRβ repertoire of CD4 Tconv cells in obese VAT
• Dysglycemia prompts an increased recirculation of PD-1+CD4 Tconv cells to the bloodstream
• PD-1+CD4 Tconv cells accumulate in the liver of patients with dysglycemic

## Graphical abstract

**Figure:**
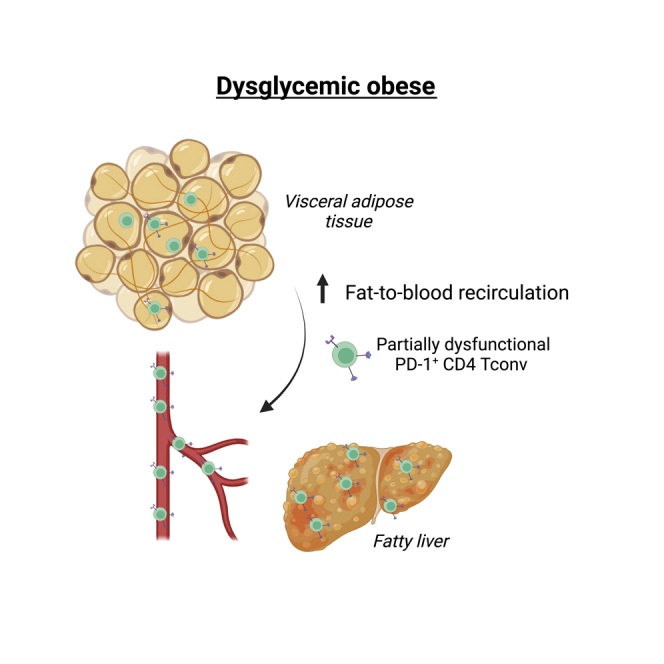

## Introduction

Insulin resistance (IR) is a pathological condition associated with obesity characterized by a reduced responsiveness of insulin-sensitive tissues -such as visceral adipose tissue (VAT), liver, and skeletal muscles-to the effects of insulin.^1^^,^^2^ The excessive accumulation of lipids in adipocytes can trigger the tissue recruitment of immune cells that differentiate into proinflammatory cytokine-producing cells and disrupt the insulin signaling pathway.^3^ The inability of pancreatic β-cells to compensate for the loss of insulin sensitivity results in dysglycemia, which progresses to the development of type 2 diabetes (T2D).^4^

In preclinical models of obesity, the dynamics of immune cells in VAT were studied. CD8 T cells have been described to precede the recruitment of macrophages into the VAT and contribute to IR by recognizing stress markers on the surface of adipocytes.^5^^,^^6^ Once in the tissue, macrophages switch to proinflammatory M1 cells, which have the ability to inactivate insulin receptor substrates.^7^^,^^8^^,^^9^ In addition, resident CD4 T cells shift from a T helper 2 (Th2) to a T helper 1 (Th1) phenotype and contribute to IR in both animal models of obesity^10^^,^^11^ and humans.^12^ More recent reports have revealed a senescent/exhausted phenotype of VAT-infiltrating CD4 T cells, which maintain the ability to release proinflammatory molecules and promote dysglycemia.^13^^,^^14^^,^^15^^,^^16^ Notably, CD4 T cells are resistant to cytokine-mediated suppression^13^ and have a restricted T cell receptor (TCR) repertoire.^10^^,^^17^ Collectively, these findings suggest that chronic stimulation by unknown cognate antigens may be responsible for the phenotypic and functional specialization of CD4 T cells in obese VAT, which eventually leads to the development of dysglycemia.

Currently, there is limited understanding of tissue-specific characteristics of VAT-derived CD4 T cells and their role in causing dysglycemia in human obesity. In this study, we have identified a subset of PD-1-expressing CD4 conventional T cells (CD4^+^CD25^−^FoxP3^-^; Tconv) which are decreased in the VAT of obese patients with concomitant dysglycemia (OB-Dys) compared to those without dysglycemia (OB-ND) and display a transcriptomic and functional profile compatible with partially dysfunctional cells. Our findings indicate that PD-1^+^CD4 Tconv cells from OB-Dys are non-clonally expanding cells that circulate from VAT to peripheral blood and are enriched in the liver of patients with dysglycemic with non-alcoholic steatohepatitis (NASH).

## Results

### Dysglycemia is associated with reduced frequency of PD-1^+^CD4 Tconv cells with an effector memory profile in obese visceral adipose tissue

We first determined the phenotypic profile of VAT-derived CD4 T cells in OB-Dys using an 11-color flow cytometry panel. The stromal vascular fraction (SVF) was isolated from the VAT of OB-ND and OB-Dys individuals collected during bariatric surgery. In terms of age, BMI, fat mass, and kidney function, OB-ND and OB-Dys groups were homogeneous (Table 1). As expected, individuals with OB-Dys demonstrated altered glycemic control, as evidenced by increased levels of HOMA-IR, fasting glucose, and A1c. We observed a higher percentage of males in the OB-Dys group, which is consistent with the increased prevalence of T2D in obese males.^18^

**Table 1:** Patient characteristics

	OB-ND (n = 16)	OB-Dys (n = 20)	p value
Age (years)	39.0 (18.0–45.4)	46.6 (25.4–51.4)	ns
Sex (F/M)	17/4	9/11	∗
Fasting glucose (mg/dL)	85.5 (75–89.25)	102 (84–123)	∗∗∗∗
A1c (mmol/mol)	36 (31–36)	44.2 (36–51)	∗∗∗∗
BMI (kg/cm^2^)	40.6 (35.4–43.2)	44.85 (34.1–46.0)	ns
Fat Mass (%)	45.8 (32.1–49.6)	44.3 (32.3–46.6)	ns
HOMA-IR	2.0 (0.8–2.7)	4.07 (2.4–8.1)	∗
Total cholesterol (mg/dL)	186 (126–218)	201 (128–206)	ns
Triglycerides (mg/dL)	113 (55–177)	132 (70–170)	ns
eGFR (mL/min/1.73 m^2^)	111 (91–122)	108 (94.8–114.8)	ns
AST (U/L)	22 (17.5–24.5)	26.5 (19.0–31.3)	ns

Values are shown as median value with interquartile range. Statistical analysis has been carried out with unpaired Mann Whitney test for all variables except for sex (Fisher’s exact test). ∗p < 0.05, ∗∗p < 0.01, ∗∗∗p < 0.001, ∗∗∗∗p < 0.0001. A1c, glycated hemoglobin; BMI, body mass index; HOMA-IR, Homeostasis Model Assessment of Insulin Resistance; eGFR, estimated glomerular filtration rate; AST, Aspartate Aminotransferase.

**Figure 1:**
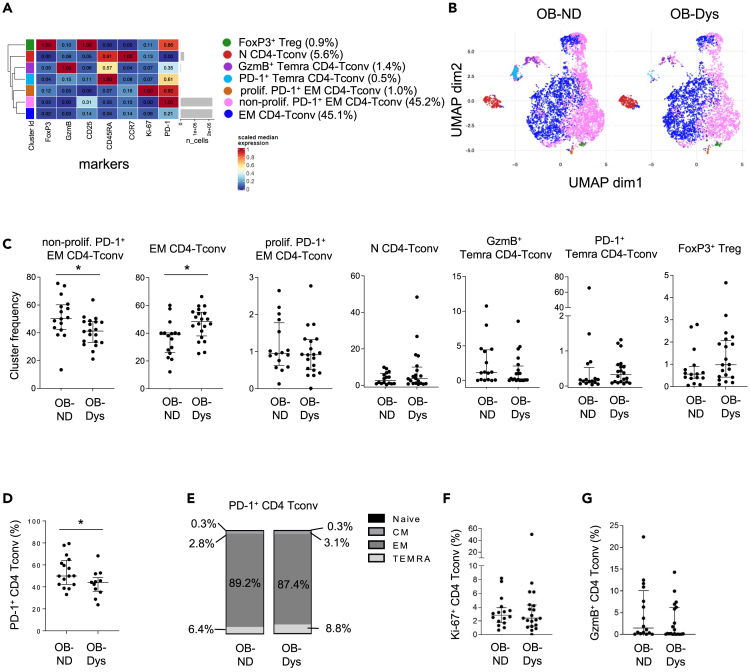
PD-1+ CD4 Tconv are reduced in the VAT of OB-Dys (A) Heatmap showing color-coded marker expression of each cluster identified after gating on CD4 T cells in the VAT of OB-ND (n = 16) and OB-Dys (n = 20). Gray bars indicate the number of cells per cluster. (B) Uniform Manifold Approximation and Projection (UMAP) visualization of the CD4 T cell clusters identified in the VAT of OB-ND and OB-Dys. (C) Frequency of cell clusters identified within the CD4 T cell subset through unsupervised analysis of flow cytometry data (FlowSOM algorithm). Bars in dot plots represent median values with interquartile range. OB-ND (n = 16) and OB-Dys (n = 20). Statistical analysis: Mann-Whitney U test. (D) Frequency of PD-1^+^CD25^−^FoxP3^-^ CD4 T conventional cells (Tconv) obtained using supervised flow cytometry analysis. Bars in dot plots represent median values with interquartile range. OB-ND (n = 16) and OB-Dys (n = 20). Statistical analysis: Mann-Whitney U test. (E) Median frequency of naive (CCR7^+^CD45RA^+^), central memory (CM, CCR7^+^CD45RA), effector memory (EM, CCR7^−^CD45RA^−^) and terminally differentiated effector memory cells (TEMRA, CCR7^−^CD45RA^+^) on PD-1^+^CD4 Tconv cells from VAT of OB-ND (n = 16) and OB-Dys (n = 20). Statistical analysis: Mann-Whitney U test. (F) Median frequency of Ki-67^+^ and (G) Granzyme B^+^ (GzmB) cells on PD-1^+^ CD4 Tconv cells from VAT of OB-ND (n = 16) and OB-Dys (n = 20). Data are presented as the median + IQR. Statistical analysis: Mann-Whitney U test. ∗p < 0.05, ∗∗p < 0.01, ∗∗∗p < 0.001, ∗∗∗∗p < 0.0001.

Unsupervised analysis of flow cytometry data led to the identification of 7 cell clusters in the VAT (Figures 1A and 1B). Hierarchical clustering of these subsets grouped patients with prediabetes with those with type 2 diabetes, indicating the preferential clusterization of OB-Dys VATs over OB-ND (Figure S1). Two cell clusters, referred to as effector memory (EM) CD4 Tconv and non-proliferating PD-1^+^ EM CD4 Tconv, accounted for over 90% of the phenotype observed in the VAT and could be distinguished based on the expression of the PD-1 marker (Figures 1A and 1B). While non-proliferating PD-1^+^ EM CD4 Tconv cells, which express low levels of the proliferation marker Ki-67 and the cytotoxic serine protease Granzyme B (GzmB), were found to be reduced in OB-Dys (Figure 1C), EM CD4 Tconv cells were consensually increased (Figure 1C). Supervised analysis of flow cytometry data (gating strategy shown in Figure S2) confirmed the decreased frequency of PD-1^+^ cells within the CD4 Tconv compartment (CD3^+^CD4^+^CD25^−^FoxP3^-^) in OB-Dys compared to OB-ND (Figure 1D). A similar frequency of Annexin+ PD-1^+^ cells indicated that the reduction was not due to increased apoptosis (Figure S3). The differentiation profile of PD-1^+^CD4 Tconv cells was similar in the two groups, with the majority of cells displaying an effector memory phenotype (CD45RA^−^CCR7^-^) and <0.5% of naive cells (CD45RA^+^CCR7^+^) (Figure 1E). No difference in the frequency of Ki-67^+^ (Figure 1F) and GzmB^+^ (Figure 1G) cells was evident between the two groups of individuals.

Overall, these findings indicate that a subset of PD-1-expressing CD4 Tconv cells with a predominant EM phenotype is reduced in the VAT of OB-Dys compared to OB-ND.

### Visceral adipose tissue-derived PD-1+CD4 Tconv cells from obese patients with dysglycemia display transcriptomic and functional characteristics of partially dysfunctional cells

**Figure 2:**
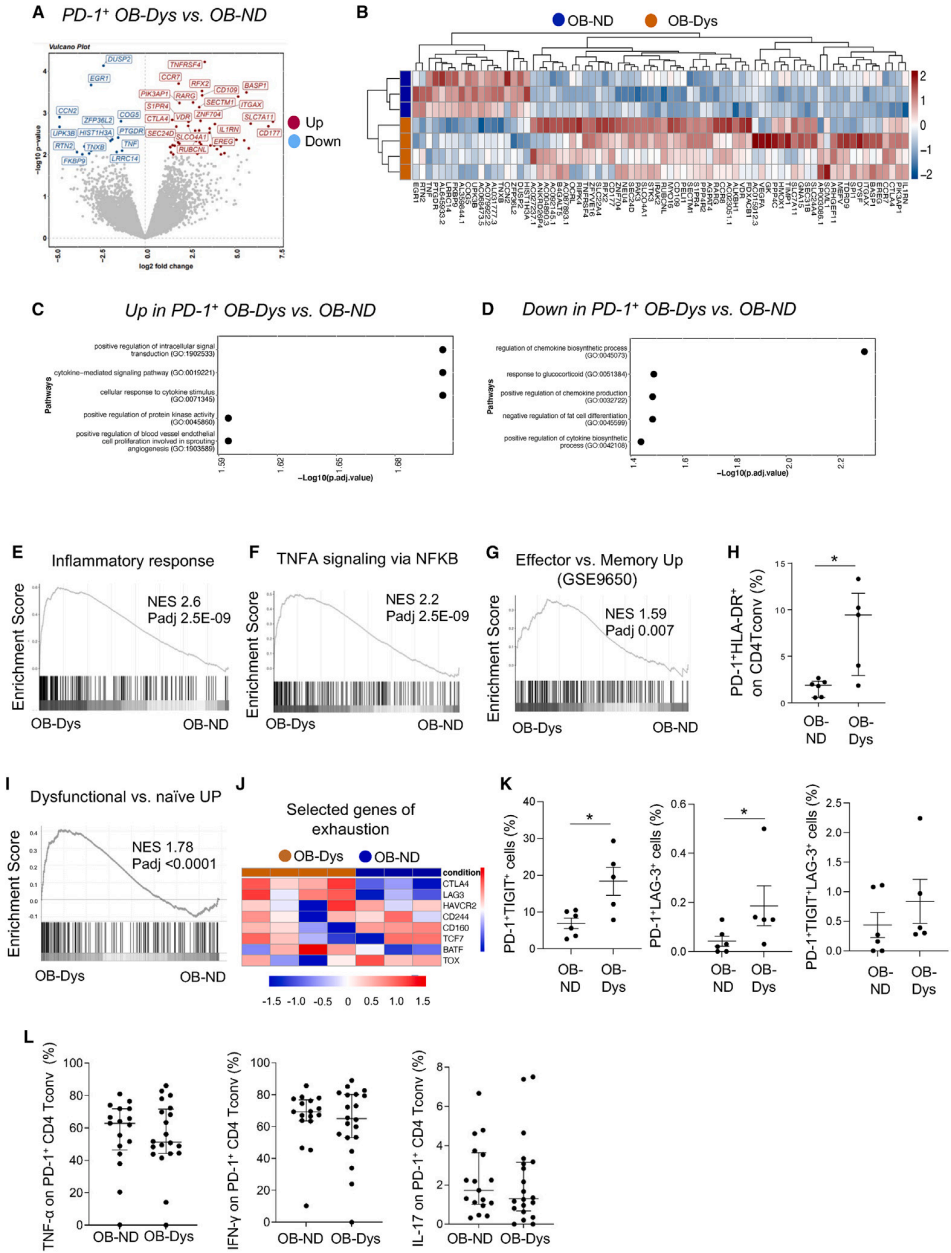
The transcriptional profile of PD-1^+^CD4 Tconv from the VAT of OB-Dys reveals effector and dysfunctional characteristics (A) The Volcano plot shows genes significantly downregulated (blue) and upregulated (red) in PD-1^+^Tconv cells from OB-Dys (n = 4) compared to OB-ND (n = 3). (B) Heatmap representing DEGs in OB-Dys (orange, n = 4) compared to OB-ND (blue, n = 3). The dendrograms show hierarchical clustering of DEGs and samples. Gene-ontology biological processes enriched in genes upregulated C) and downregulated D) in VAT-derived PD-1^+^CD4 Tconv cells from OB-Dys (n = 4) vs. OB-ND (n = 3). (E) Gene set enrichment analysis (GSEA) of the “Inflammatory response” and F) “TNFA signaling via NFKB” signatures (MSigDB Hallmark 2020) in our dataset (OB-Dys = 4, OB-ND = 3). (G) GSEA of the genes upregulated in effector vs. memory cells (GSE9650 dataset) (OB-Dys = 4, OB-ND = 3). (H) Frequency of PD1^+^HLA-DR^+^ cells on CD4 Tconv cells (OB-ND, n = 6; OB-Dys, n = 5). Statistical analysis: Mann-Whitney U test. (I) GSEA of the genes upregulated in melanoma-infiltrating dysfunctional CD8 T cells^20^ (OB-Dys = 4, OB-ND = 3). (J) Heatmap showing expression level (Log2 RPKM) of selected exhaustion-associated genes (OB-Dys = 4, OB-ND = 3). (K) Frequency of PD1^+^TIGIT^+^, PD1^+^LAG-3^+^ and PD1^+^TIGIT^+^LAG-3^+^ cells on CD4 Tconv cells in VAT of OB-ND (n = 6) and OB-Dys (n = 5). (L) Frequency of IFN-ɣ, TNF-α and IL-17-producing PD-1^+^CD4 Tconv cells upon PMA/Ionomycin stimulation in VAT of OB-ND (n = 16) and OB-Dys (n = 20). Data are presented as the median + IQR. Statistical analysis: Mann-Whitney U test. ∗p < 0.05, ∗∗p < 0.01, ∗∗∗p < 0.001, ∗∗∗∗p < 0.0001.

To comprehensively characterize PD-1^+^CD4 Tconv cells and discern pathways potentially implicated in VAT-specific glucose dysmetabolism, we determined the transcriptomic profile of VAT-derived PD-1^+^CD4 Tconv cells from OB-ND and OB-Dys. We focused our analysis on the PD-1^+^ compartment guided by our transcriptomic findings that revealed a diminished relevance of the PD-1^-^ cell subset in the context of dysglycemia. This was substantiated by the relatively low number of differentially expressed genes (DEGs) in OB-Dys compared to OB-ND (n = 33) and the absence of relevant functional enrichments, as observed through both EnrichR and GSEA platforms (data not shown). PD-1^+^CD4 Tconv from patients with OB-Dys showed 58 genes upregulated and 19 downregulated compared to OB-ND (Figures 2A and 2B). The complete list of DEGs is provided in Table S1. Relevant DEGs include molecules involved in immune responses, such as *IRAK2*, *TNFRSF4*, *IL1RN*, and *PTGDR* (Figure 2A). PD-1^+^CD4 Tconv cells from OB-Dys upregulated genes related to cytokine responses (Figure 2C), while downregulating pathways associated with glucocorticoid signaling and with the inhibition of fat differentiation (Figure 2D). Furthermore, PD-1^+^CD4 Tconv cells from OB-Dys displayed a proinflammatory phenotype as indicated by the enrichment of gene signatures of “inflammatory response” (Figure 2E) and “TNFA signaling via NFKB” (Figure 2F). Consistent with this observation, the signature of effector cells was found enriched in PD-1^+^CD4 Tconv cells from OB-Dys (Figure 2G) as well as an increased frequency of activated PD-1^+^HLA-DR^+^ CD4 Tconv cells was observed in OB-Dys VAT (Figure 2H).^19^

Notably, the signature of tumor-infiltrating dysfunctional T cells was also enriched in VAT-derived PD-1^+^CD4 Tconv cells from OB-Dys (Figure 2I). PD-1+CD4 Tconv cells from OB-Dys exhibited the expression of T cell exhaustion markers, including CTLA-4 and LAG-3, which were absent in patients with OB-ND (Figure 2J). Nonetheless, other exhaustion-associated inhibitory receptors and transcription factors such as *TCF7*, *BATF*, and *TOX* displayed comparable expression levels in both patient groups (Figure 2J). Additionally, an elevated frequency of PD-1^+^TIGIT^+^ and PD-1^+^LAG-3^+^ co-expressing CD4 Tconv cells was observed in OB-Dys (Figure 2K, left and middle panels), while the frequency of CD4 Tconv cells expressing multiple inhibitory receptors (i.e., PD-1+TIGIT+LAG-3+, Figure 2K, right panel) remained similar between the two groups. Markers of senescence remained unaltered in the presence of dysglycemia, as demonstrated by comparable expression levels of genes associated with senescence (Figure S4A)^21^ as well as an equivalent frequency of CD57+PD-1+CD4 Tconv cells between OB-Dys and OB-ND (Figure S4B) Notably, no difference in the frequency of IFN-ɣ, TNF-α, and IL-17-producing PD-1^+^CD4 Tconv cells between VATs could be detected following PMA/Ionomycin stimulation (Figure 2L), indicating that the engagement of the TNFA/effector signaling pathway by VAT-derived PD-1^+^ cells does not result in increased release of proinflammatory soluble molecules.

Two-hundred twenty-seven genes were found to be positively correlated with HOMA-IR levels in PD-1^+^CD4 Tconv cells. These genes were enriched in pathways associated with inflammatory responses, including TNF-ɑ and Interferon-ɑ responses (Figure S5A; Table S2). Among those genes, *TNFRSF4* and *HMOX1*, which are DEGs between PD-1^+^CD4 Tconv cells of OB-ND and OB-Dys, have been previously described to be mechanistically linked to IR^22^^,^^23^ (Figure S5B). However, protein content of *TNFRSF4* and *HMOX1* was similar between OB-ND and OB-Dys in PD-1^+^CD4 Tconv cells (Figure S5C).

These data indicate that VAT-derived PD-1^+^CD4 Tconv cells from OB-Dys are heterogeneous and display a transcriptional and functional profile compatible with partially dysfunctional cells.

### Visceral adipose tissue-derived CD4 Tconv cells are a heterogeneous population, with enrichment in virus-specific cells within the public clonotypes

As PD-1 can mark antigen-specific T cells^24^^,^^25^^,^^26^ and clonality of VAT-derived T cells has been described in preclinical models of obesity,^17^^,^^27^ we wondered whether PD-1^+^CD4 Tconv cells undergo clonal expansion toward specific antigens in the VAT of OB-Dys. To ascertain the extent of clonality under physiological conditions, for these experiments we included a cohort of n = 4 non-obese non-diabetic donors undergoing living kidney donation named lean controls (LC). Sequencing of the T cell receptor β-chain complementarity determining region 3 (TCRβ CDR3) of PD-1^+^ and PD-1^-^ CD4 Tconv cells from VAT of OB-ND, OB-Dys, and LC revealed a similar distribution of the TCR-V_β_ gene families suggesting a superimposable composition of TCRβ repertoires among the 3 groups (Figure S6). This was confirmed by the comparable richness of the TCRβ repertoire among the three groups as indicated by the Shannon’s diversity index (Figure S7). These data are further supported by the similar frequency of CD137^+^ PD-1^+^CD4 Tconv, which identify recently antigen-activated T cells,^28^^,^^29^ in patients with OB-ND and OB-Dys (Figure S8).

**Figure 3:**
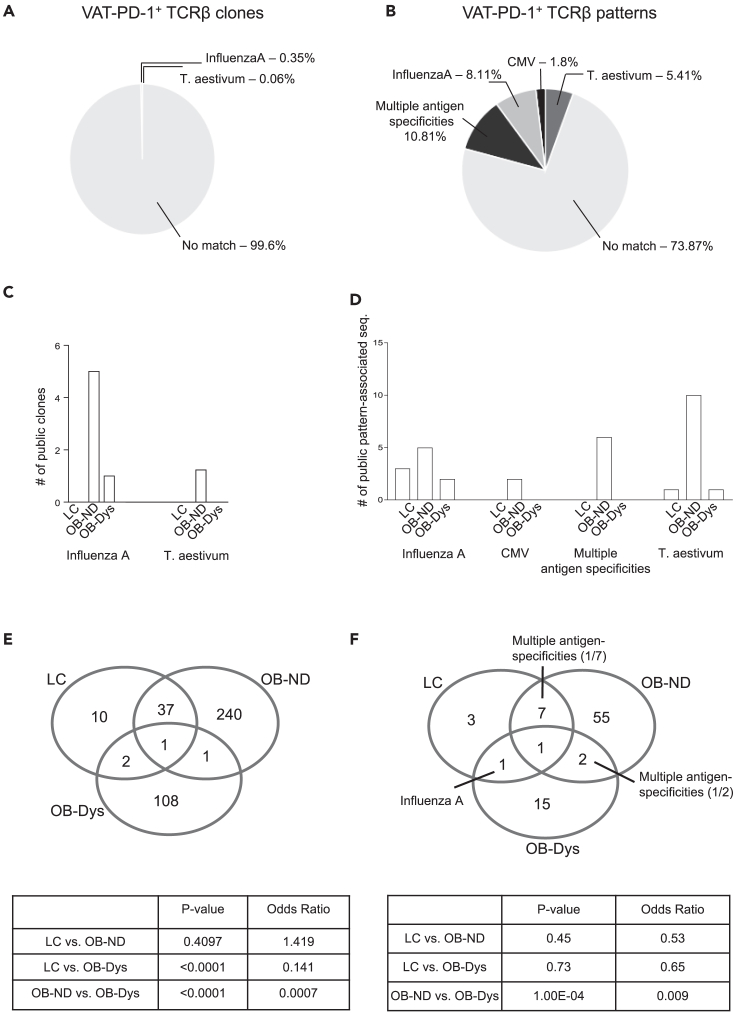
The TCRβ repertoire of PD-1^+^ CD4 Tconv cells in VAT is altered in OB-Dys and is characterized predominantly by virus-specific public clonotypes (A) Pie chart representing the frequency of clones with a match for antigens annotated in the VDJ database. (B) Pie chart representing the frequency of patterns - obtained using the GLIPH2 algorithm - with a match for antigens annotated in VDJdb. (C) Number of clones or (D) pattern-associated sequences with a unique match in VDJdb. In “multiple antigen specificities" bars indicate the number of pattern-associated sequences matching with multiple antigens. (E) Venn diagrams showing the number of clones and (F) patterns shared among LC, OB-ND and OB-Dys individuals in VAT-derived PD-1^+^CD4 Tconv cells. Matches with public clones annotated in VDJdb are reported. Exact Fisher’s test has been adopted to evaluate the association between shared and unshared clones and patterns for each comparison (LC vs. OB-ND, LC vs. OB-Dys, OB-ND vs. OB-Dys). LC (n = 4), OB-ND (n = 6) and OB-Dys (n = 7).

In the attempt to infer the antigen specificity of clonotypes identified in VAT-derived PD-1^+^CD4 Tconv from OB-Dys, we investigated whether public clones contributed to their TCRβ repertoire. At first, we searched in our dataset for the presence of TCRs with known antigen specificity reported in the VDJdb database. Results showed that 0.4% amino acid sequences of PD-1^+^CD4 Tconv cells from 4/7 OB-Dys, 3/6 OB-ND, and 2/4 LC matched with public clonotypes responding to viral, pathogen, and wheat antigens annotated in VDJdb (Figure 3A), with specificities toward Influenza A and T. Aestivum. To increase the chance to find public clonotypes in VDJdb, we exploited the GLIPH2 algorithm that clusters together TCR sequences with similar motifs (called patterns) which are predicted to bind the same antigens [32]. Amino acid sequences that did not meet GLIPH2 criteria (see STAR methods section) were excluded from the analysis. Twenty-six percent of PD-1^+^CD4 Tconv patterns identified in the VAT were public (Figure 3B), with specificities toward Influenza A, T. aestivum and CMV. A total of 10.81% of patterns showed specificities for multiple antigens, including Influenza A, CMV, HIV-1, and the tumor antigen NY-ESO1. The distribution of public clonotypes (Figure 3C) and public pattern-associated sequences (Figure 3D) among the three groups is shown. Similar matches of TCRβ clones could be observed in VAT-derived PD-1^-^ cells (Figure S9A), with 24.4% of PD-1^-^ TCRβ patterns being public and specific for Influenza A, T. aestivum, CMV, RSV and HIV-1 (Figure S9B). Notably, the VAT of obese patients showed a trend of enrichment in CD4 Tconv public clonotypes compared to LC, regardless of PD-1 expression or diabetes status (Figures 3C, 3D, and S9C).

These data indicate that VAT-derived CD4 Tconv cells exhibit a predominant virus-specificity among public clonotypes, irrespective of PD-1 expression or diabetes status.

### The T cell receptor β-chain repertoire of visceral adipose tissue-derived CD4 Tconv cells is selectively altered in obese patients with dysglycemia

Although VAT-derived PD-1^+^CD4 Tconv cells demonstrate a polyclonal constitution in the context of obesity, we sought to elucidate whether the presence of dysglycemia engenders a distinctive profile in the TCRβ repertoire. We specifically focused on clones that exhibit presence in at least two individuals, effectively excluding private clonotypes from the analysis. Notably, discernible patterns emerge as TCRβ clones identified within the OB-Dys subgroup exhibit a proclivity to be intra-patient common (Fisher’s exact test, OB-Dys vs. LC and OB-Dys vs. OB-ND, p value <0.0001), while their convergence with the lean control (LC) (OR = 0.141) and OB-ND (OR = 0.0007) cohorts is infrequent (Figures 3E and S10). This observation underscores the marked divergence in the TCRβ repertoire of VAT-derived PD-1^+^CD4 Tconv cells as dysglycemia becomes a feature. In contrast, TCRβ clones tend to be casually distributed between the LC and OB-ND groups (Fisher’s exact test, p value 0.4). TCRβ patterns identified by the GLIPH2 algorithm differed significantly between OB-ND and OB-Dys, while no significant difference was observed in LC vs. OB-ND and LC vs. OB-Dys (Figure 3F). Similar trends were also observed in the PD-1^-^ compartment (Figure S10).

Overall, these data suggest that an altered composition of the TCRβ repertoire of VAT-derived CD4 Tconv cells can be observed in OB-Dys regardless of PD-1 expression.

### PD-1^+^CD4 Tconv cells from obese patients with dysglycemia show increased recirculation of clones with a non-restricted T cell receptor β-chain repertoire from visceral adipose tissue to PB

**Figure 4:**
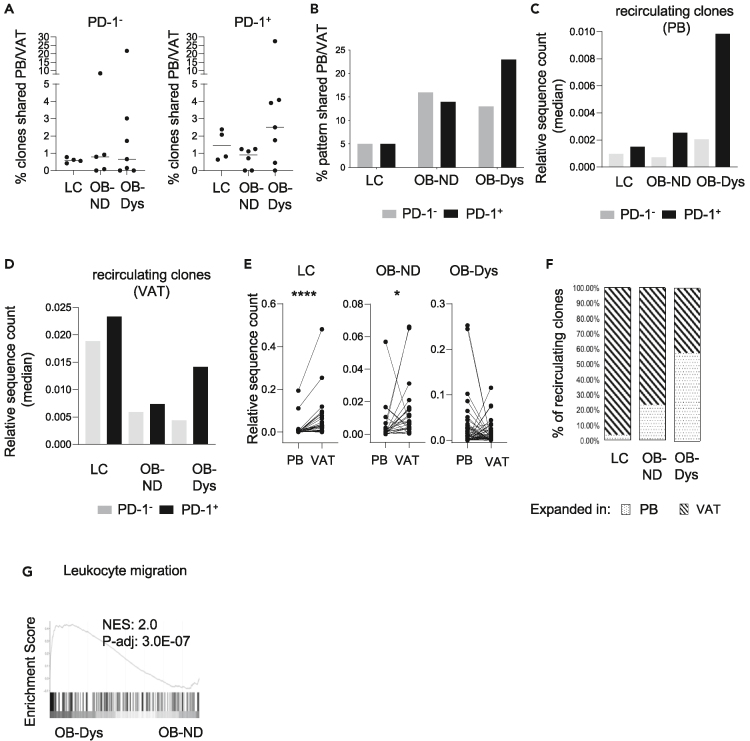
Increased recirculation of non-restricted TCR*β* clones of PD-1+ CD4 Tconv from VAT to PB is observed in OB-Dys (A) Frequency of TCR*β* amino acid sequences shared between PB and VAT of LC (n = 4), OB-ND (n = 6) and OB-Dys (n = 7). Data are presented as median. Statistical analysis: Kruskal-Wallis test. (B) Percentage of recirculating patterns in PB and VAT in PD1- (gray bars) and PD1+ (black bars) CD4 Tconv. (C) Median relative sequence count of recirculating PD1- (gray bars) and PD1+ (black bars) CD4 Tconv TCR*β* clones in PB. (D) Median relative sequence count of recirculating PD1- (gray bars) and PD1+ (black bars) CD4 Tconv cells in VAT. (E) Relative sequence count of recirculating PD1+CD4 Tconv cells in PB and VAT of LC, OB-ND and OB-Dys. Statistical analysis: Wilcoxon matched pairs signed ranked test. (F) Stacked bar plots showing the percentage of recirculating clones of LC, OB-ND and OB-Dys whose relative sequence count is higher in VAT (stripes) or in PB (dots). Data represented from Figures 4A to 4F have been obtained from LC (n = 4), OB-ND (n = 6) and OB-Dys (n = 7). (G) GSEA of the “leukocyte migration” signature in PD-1+CD4 Tconv cells of OB-Dys (n = 4) vs. OB-ND (n = 3). ∗p < 0.05, ∗∗p < 0.01, ∗∗∗p < 0.001, ∗∗∗∗p < 0.0001.

To explain why a shortage of partially exhausted PD-1^+^CD4 Tconv was observed in the VAT of OB-Dys, we tested the hypothesis that this cell subset develops the ability to recirculate from VAT to PB when dysglycemia occurs. A clonal tracking analysis was performed to determine the frequency of clones shared between PB and VAT within the same individuals referred to as “recirculating clones.” The median percentage of recirculating clones was higher in the VAT of OB-Dys compared to OB-ND and LC in PD-1^+^, but not PD-1^-^, CD4 Tconv (Figure 4A). This observation was corroborated by two key lines of evidence: firstly, the TCRβ patterns of PD-1^+^CD4 Tconv cells from patients with OB-Dys exhibited the most pronounced rate of recirculation between PB and VAT, an observation not mirrored by their PD-1^-^ counterparts (Figure 4B); secondly, it was evident that recirculating PD-1^+^CD4 Tconv clones from OB-Dys were expanded in the PB (Figure 4C). Notably, recirculating clones from both the PD-1^+^ and PD-1^-^ compartments were found to be less expanded in obese VATs compared to LC (Figure 4D), suggestive of a non-clonal TCR repertoire of recirculating clones in obesity regardless of diabetes status.

To discern the trajectory (VAT to PB or PB to VAT) of clone recirculation, we ascertained whether the clones were preferentially expanded within the VAT or PB compartments. The results revealed a distinctive pattern: while 97% and 77% of recirculating PD-1^+^ CD4 Tconv cells from LC and OB-ND, respectively, were expanded in the VAT, the majority (58%) of recirculating clones from OB-Dys exhibited expansion within the PB compartment (Figures 4E and 4F). This underscores a shift in the directionality of recirculating clones within the context of OB-Dys, deviating from the conventional PB to VAT trajectory observed in physiological/normoglycemic conditions. A similar inversion of clones’ expansion could be observed in the PD-1^-^ counterpart (Figure S11A), with 96% and 72% clones expanded in VAT of LC and OB-ND, respectively, and 53% expanded in PB of OB-Dys (Figure S11B). Further evidence of increased PD-1^+^CD4 Tconv cell recirculation in OB-Dys was provided by the enrichment of the “leukocyte migration” signature in OB-Dys compared to OB-ND (Figure 4G). Of note, the expression of *S1PR4*, which regulates T cell migration,^30^ could be found among upregulated DEGs in PD-1^+^ cells from OB-Dys compared to OB-ND (Figures 2A and 2B) and positively correlated with HOMA-IR levels (Table S2).

All together these data indicate that dysglycemia in human obesity is associated with increased recirculation of PD-1^+^CD4 Tconv cells with unrestricted TCRβ repertoire from VAT to PB.

### Circulating PD-1^+^CD4 Tconv cells with altered migratory potential are reduced in the circulation of obese patients with dysglycemia and accumulate in the liver of patients with dysglycemic non-alcoholic steatohepatitis

**Figure 5:**
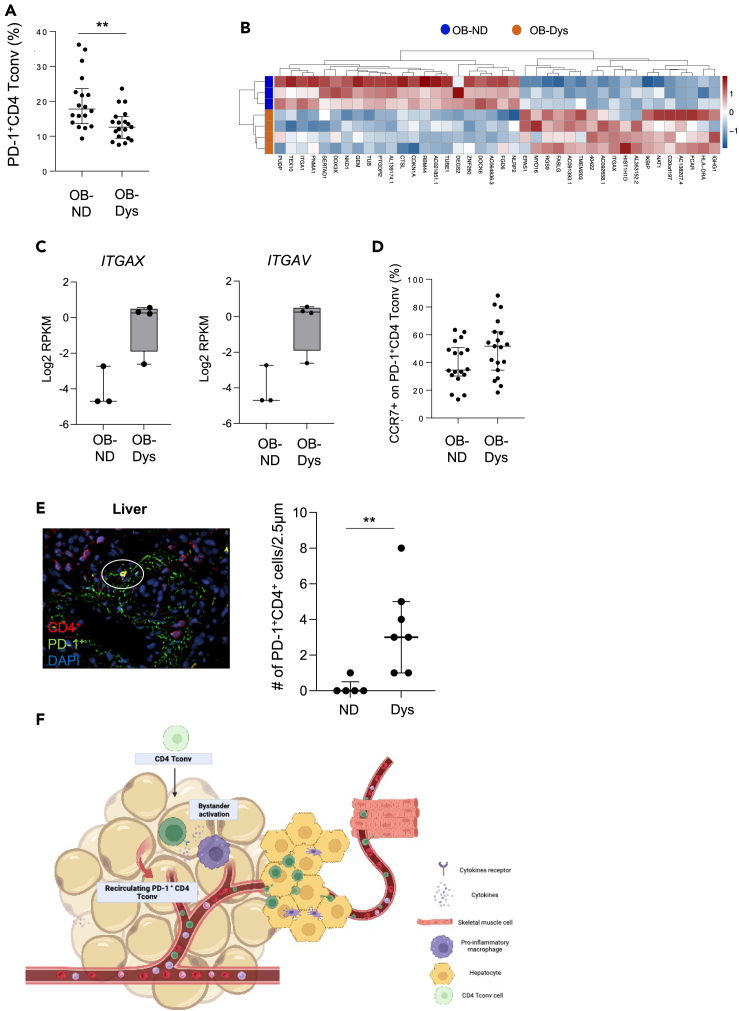
PD-1^+^CD4 Tconv cells are reduced in PB of OB-Dys and enriched in the liver of patients with NASH with dysglycemia (A) Frequency of PD-1+CD4 Tconv in PB of OB-ND (n = 18) and OB-Dys (n = 20). Statistical analysis: Mann-Whitney test. (B) Heatmap representing DEGs between PB-derived PD-1+CD4 Tconv from OB-Dys (orange, n = 4) vs. OB-ND (blue, n = 3). The dendrograms show hierarchical clustering of DEGs and samples. (C) mRNA expression levels of the integrins alpha X chain *ITGAX* and Alpha V chain *ITGAV* in PB-derived PD-1+CD4 Tconv from OB-ND (n = 3) and OB-Dys (n = 4). (D) Frequency of CCR7+ cells on PD-1+ CD4 Tconv from PB of OB-ND (n = 18) and OB-Dys (n = 20). Statistical analysis: Mann-Whitney test. (E) Immunofluorescence showing PD-1, CD4 and DAPI in sections of paraffin-embedded human liver from a representative patient with NASH. Double-positive cells CD4+PD1+ cells are shown in the circle (left panel). Number of CD4+PD-1+ cells per HPF (40X) detected in the liver of patients with NASH with (n = 7) and without (n = 5) dysglycemia (right panel). Data are presented as the median + IQR. Statistical analysis: Mann-Whitney test. (F) Graphical representation of reported data: PD-1+CD4 Tconv cells localized in VAT of obese patients with dysglycemia are locally induced to differentiate into partially dysfunctional cells undergoing bystander cytokine-mediated activation. Recirculating PD-1+CD4 Tconv cells acquire a migratory profile, leave the VAT and migrate to other insulin-sensitive tissues, such as liver and possibly muscles, where they can contribute to local inflammation leading to IR. ∗p < 0.05, ∗∗p < 0.01, ∗∗∗p < 0.001, ∗∗∗∗p < 0.0001.

The reduced frequency of PD-1^+^CD4 Tconv cells in VAT of OB-Dys was expected to be associated with their accumulation in PB. However, the frequency of PD-1^+^CD4 Tconv cells was found to decrease in PB of OB-Dys compared to OB-ND (Figure 5A) and not imputable to apoptosis (Figure S12). The transcriptomic analysis of PB-derived PD-1^+^CD4 Tconv cells showed differential expression of 40 genes (18 upregulated and e 22 downregulated) between OB-Dys and OB-ND (Figure 5B), with the deregulation of the “integrin family cell surface interaction” pathway (NCI Nature 2016). An altered integrin expression profile was evident in PD-1^+^CD4 Tconv cells from OB-Dys, which included higher expression levels of *ITGAX* – enriched in T cells with high migratory potential^31^ – and *ITGAV* – associated with cancer progression^32^ – (Figure 5C), and lower expression of *ITGA1*, tissue-resident memory T cell marker^33^ (data not shown). Furthermore, a trend of increased protein expression of the chemokine receptor CCR7, regulator of T cell trafficking and recruitment into inflamed tissues^34^^,^^35^ – was observed in OB-Dys compared to OB-ND (p = 0.08) (Figure 5D), while the proportion of CCR7+ cells, i.e., naive and central memory PD-1+ CD4 Tconv cells, remain unchanged (Figure S13). Notably, cells co-expressing PD-1 and CD4 were found to be enriched in the liver of patients with NASH and concomitant dysglycemia compared to patients with normoglycemic NASH (Figure 5E).

Collectively, these findings underscore the linkage between dysglycemia and a diminished prevalence of PB-derived PD-1^+^CD4 Tconv cells characterized by augmented migratory potential. Furthermore, these results concomitantly highlight the accumulation of PD-1^+^CD4^+^ cell populations within the hepatic milieu of patients with dysglycemic NASH.

## Discussion

The role of CD4 T cells in the initiation and maintenance of low-grade inflammation at the target site of inflammation of human obesity, i.e., the VAT, is still controversial, as it is their contribution to the development of T2D.^10^^,^^11^^,^^12^^,^^14^^,^^16^ This study, by evaluating CD4 Tconv phenotype, RNA transcriptome, and receptor clonality in the VAT and PB of patients with and without dysglycemia, attempts to elucidate the link between adaptive immunity and T2D.

While Th1 cells for a long time have been imputed as drivers of tissue inflammation in obesity, more recent studies have found that CD4 T cells from obese VAT are functionally exhausted, with PD-1^+^ cells endowed with senescent characteristics.^14^^,^^16^ We hereby demonstrate that PD-1^+^CD4 Tconv cells are reduced in the VAT of obese patients with dysglycemia and recirculate into the bloodstream. We found that PD-1-expressing CD4 Tconv cells in the VAT of OB-Dys are a heterogeneous population with transcriptomic and functional characteristics compatible with partially dysfunctional cells. T cell exhaustion is a differentiation state occurring in the presence of persistent chronic TCR stimulation with dysfunctional cells expressing arrays of inhibitory molecules, distinctive patterns of transcription factors, and impaired effector molecules. Recent studies reported a gradient of exhaustion,^36^ with dysfunctional T cells being a heterogeneous population that is part of a wide differentiation spectrum, spanning from transitional, through early dysfunctional, toward highly dysfunctional T cells.^20^^,^^37^^,^^38^ Dysfunctional cells exhibit a spectrum that encompasses the retention of certain effector functionalities,^39^^,^^40^ manifestation as early-stage or partially exhausted states,^20^ which concurrently manifests an intermediate level of the TOX transcription factor.^41^ Within the VAT microenvironment of OB-Dys, PD-1^+^CD4 Tconv cells: (i) manifest a transcriptomic profile that bridges effector and dysfunctional phenotypes, (ii) following stimulation, do not show increased proinflammatory cytokine synthesis, (iii) demonstrate co-expression of TIGIT or LAG-3, but not multiple inhibitory receptors, and (iv) exhibit an intermediate expression level of TOX. Collectively, this evidence implies the likelihood of these cells being in the earlier stages of partial dysfunction. Such a profile could correspond to cells that are less sensitive to modulation or immunosuppression as previously shown by our group on bulk CD4 Tconv cells from OB-Dys.^13^ Single cell resolution of the PD-1^+^CD4 Tconv cell transcriptome will help elucidate the heterogeneity of this cell subset. However, one could hypothesize that localized soluble factors and cell-to-cell interactions ultimately intercede with proinflammatory/effector signaling pathways, thereby fostering the development of a subset of PD-1^+^CD4 Tconv cells with a partially exhausted phenotype. Also, it cannot be excluded that the PD-1 signaling is not fully functional in this context, thus resulting in the freezing of PD-1^+^CD4 Tconv cells in a transitional state of differentiation. The pivotal question revolves around whether this partial impairment of PD-1^+^ CD4 Tconv cells in OB-Dys is beneficial or impedes the resolution of chronic low-grade inflammation in adipose tissue. Our hypothesis posits that PD-1^+^ CD4 Tconv cells may undergo partial exhaustion in the context of dysglycemia, acting as a physiological feedback mechanism to attenuate metabolic inflammation sustained by macrophages. However, it is plausible that unresolved T cell stimulation through the TCR could result in a lack of inhibitory signals to macrophages, potentially perpetuating a sustained inflammatory response.

Adipose tissue serves as a reservoir for persistent pathogens, such as Mycobacterium tuberculosis, Influenza A, CMV, HIV, and pathogen-specific T cells.^42^^,^^43^^,^^44^^,^^45^^,^^46^ This evidence was confirmed by our results showing that public clonotypes, predominantly specific for viral antigens, were observed in obese VATs regardless of diabetes status. Notably, the VAT-derived TCRβ repertoire of CD4 Tconv was divergent in OB-Dys compared to the other groups. This evidence is supported by the outcomes of hierarchical clustering analysis arising from unsupervised flow cytometry data, which delineates the preferential clustering of obese VATs encompassing prediabetic and type 2 diabetic subgroups over their OB-ND counterparts. The confluence of these observations collectively suggests that dysglycemia elicits substantive modifications within the CD4 Tconv cell compartment. This study is not designed to elucidate if changes in CD4 Tconv cells determine dysglycemia or rather dysglycemia induces CD4 Tconv cell skewing; however, considering that the natural history of T2D consists in the progression from a normoglycemic state to a transient phase of prediabetes preceding the development of full-blown disease,^47^ the homogeneity of CD4 Tconv cells infiltrating the VAT in patients with dysglycemia suggests that changes of CD4 Tconv cells may occur long before the development of T2D. Furthermore, it is unlikely that the mild hyperglycemia occurring in prediabetes could explain the phenotypic and transcriptomic changes, as well as alterations of the TCR repertoire, observed in VAT-derived CD4 Tconv cells. One could speculate that gradual changes of PD-1^+^CD4 Tconv cells may occur at the target site of inflammation of obesity in response to metabolic and environmental stressors which induce a phenotypic shift toward an early dysfunctional state resulting in the engagement of a transcriptional programming of cell migration.

The phenotypic profile of PD-1^+^CD4 Tconv cells in the VAT of OB-Dys was expected to be driven by self-antigens and associated with clonal expansion, as previously suggested in different settings.^17^^,^^20^^,^^27^ While TCR clonality in PD-1^+^CD4 Tconv cells within the VAT was not observed, the presence of dysglycemia yielded distinctive outcomes: (i) an exclusive TCR repertoire configuration, (ii) increased adipose-to-circulation recirculation, and (iii) a diminished frequency yet heightened migratory potential within the PB. Furthermore, recirculating clones of PD-1^+^CD4 Tconv cells showed lower expansion in obese VAT regardless of diabetes status, suggesting that they likely undergo bystander cytokine-mediated activation. While it was not expected that the recirculation of 2% of cells from VAT to PB would lead to a clear-cut increase in PD-1^+^ CD4 Tconv cells in the PB of OB-Dys, the observed decrease was equally unexpected. This diminished frequency of PD-1^+^ CD4 Tconv cells in the PB of OB-Dys could not be explained by increased apoptosis. However, the accumulation of this cell subset in patients with NASH with dysglycemia supports the hypothesis that the bloodstream might not inherently constitute the ultimate reservoir for this cell subset and that they may further recirculate in the liver. However, additional analyses are required to substantiate this claim. Our working hypothesis underscores the conceivable role of PD-1^+^CD4 Tconv cells in effecting an inter-organ propagation of a proinflammatory milieu, thereby contributing to insulin resistance across various insulin-sensitive tissues (Figure 5F).

Reported findings indicate that dysglycemia, characterized by elevated levels of IR in human obesity, is associated with the recirculation of partially dysfunctional polyclonal PD-1^+^CD4 Tconv from VAT to PB. This study has uncovered several novel findings regarding adipose tissue biology in the context of obesity and T2D, providing the rationale for proposing T cell recirculation as the underlying mechanism of human IR. Signals released by metabolic tissues – such as peptide hormones, cytokines, exosomes, and lipids – are described to mediate inter-organ communication in the control of systemic metabolism.^48^ However, T cells have all the required features to be vehicles of metabolic inflammation as they can (i) be carriers of a proinflammatory state induced by a variety of triggers, (ii) be individually (chemo)attracted toward specific tissues, (iii) easily adapt to local environments. Here, for the first time, we describe the link between T cell recirculation and whole-body metabolism. Further investigation is needed to determine if recirculation is the mechanism underlying the development of dysglycemia or vice versa and whether altered glucose metabolism induces T cell recirculation. This newly acquired knowledge provides the rationale for further testing pharmacological compounds halting T cell recirculation,^49^^,^^50^ in the prevention or treatment of T2D.

### Limitations of the study

This study posits PD-1^+^ CD4 Tconv cells as potential mediators of insulin resistance within insulin-sensitive tissues, substantiated by evidence from a diverse range of patient cohorts. Limitations encompass the inability to chronicle events during the transition from euglycemia to dysglycemia and uncertainty regarding the direct causation of dysglycemia by these cells in type 2 diabetes. Furthermore, our observations indicate a reduction of PD-1^+^ CD4 Tconv cells in VAT of obese patients with dysglycemic and an augmentation in the liver of individuals with NASH and type 2 diabetes. Validation of these data necessitates single-cell tracking analyses in both human subjects and murine models to elucidate the causal association between PD-1^+^ CD4 Tconv cells and dysglycemia.

## STAR★Methods

### Key resources table

**Table:** 

REAGENT or RESOURCE	SOURCE	IDENTIFIER
**Antibodies**		

PE anti-human CCR7	Biolgend	Cat#353204, see Table S3 for additional details
PE-Cy7 anti-human CD127	Beckman Coulter	Cat#A64618; RRID:AB_2833031
BV786 anti-human CD137	BD	Cat#741000; RRID:AB_10913813
APC anti-human CD25	BD	Cat#340907; RRID:AB_2819021
APC-Cy7 anti-human CD25	Biolegend	Cat#356122; RRID:AB_2562488
BUV395 anti-human CD3	BD	Cat#564001; RRID:AB_2744382
PerCP anti-human CD3	Biolegend	Cat#344814; RRID:AB_10639948
BUV805 anti-human CD4	BD	Cat#612887; RRID:AB_2870176
BV711 anti-human CD4	Biolegend	Cat#317440; RRID:AB_2562912
BV510 anti-human CD45	Biolegend	Cat#304036; RRID:AB_2561940
PB anti-human CD45	Biolegend	Cat#304022; RRID:AB_2174123
PerCP-Cy5 anti-human CD45	Biolegend	Cat#304028; RRID:AB_893338
BV421 anti-human CD45RA	Biolegend	Cat#304130; RRID:AB_10900421
BV650 anti-human CD69	Biolegend	Cat#310934; RRID:AB_2563158
APC-H7 anti-human CD8	BD	Cat#560179; RRID:AB_1645481
BV605 anti-human CD8	Biolegend	Cat#344742; RRID:AB_2566513
Alexa Fluor 488 anti-human FoxP3	Biolegend	Cat#320212; RRID:AB_430886
Alexa Fluor 647 anti-human GzmB	Biolegend	Cat#515406; RRID:AB_2294995
BUV661 anti-human HLA-DR	BD	Cat#565074
PB anti-human IFNγ	Biolegend	Cat#502522; RRID:AB_893525
PE eFluor 610 anti-human Ki67	eBioscience	Cat#61-5699-42; RRID:AB_2574622
PE-CF594 anti-human LAG-3	BD	Cat#565719; RRID:AB_2869706
R718 anti-human PD-1	BD	Cat#566974; RRID:AB_2869981
PE anti-human PD-1	Invitrogen	Cat#12-2799-42; RRID:AB_11042478
PE-Cy7 anti-human PD-1	eBioscience	Cat#25-2799-42; RRID:AB_10853804
BV711 anti-human TIGIT	BD	Cat#747839; RRID:AB_2872302
BUV737 anti-human TIM-3	BD	Cat#748820; RRID:AB_2873223
PE-Cy5 anti-human TNFRSF4	BD	Cat#551500; RRID:AB_394221
BV650 anti-human TNFα	Biolegend	Cat#502938; RRID:AB_2561355
PB anti-human CD57	Biolegend	Cat#359608; RRID:AB_2562459

**Biological samples**		

Stromal Vascular Fraction	This paper	See STAR Methods
PBMC	This paper	See STAR Methods
Liver biopsies	This paper	See STAR Methods

**Critical commercial assays**		

Horizon™ Fixable Viability Stain 575V	BD	Cat#565694; RRID:AB_2869702
Fixable Viability Dye eFluor™ 506	Invitrogen	Cat#65-0866-14
BD Pharmingen™ FITC Annexin V Apoptosis Detection Kit I	BD	Cat#556547; RRID:AB_2869082
RNeasy Micro Kit	Qiagen	Cat#74004
SMART-Seq® v4 Ultra® Low Input RNA Kit for Sequencing	Takara Bio	Cat#634893
MiSeq reagent kit v3	Illumina	

**Deposited data**		

RNA-seq data	This paper	Gene Expression Omnibus: GSE237338
VDJdb	https://vdjdb.cdr3.net/	

**Software and algorithms**		

Graphpad Prism 9	Graphpad Prism 9	N/A
Flow jo version 10.8.1	Flow jo version 10.8.1	N/A
OpenCyto	Finak G, Frelinger J, Jiang W, Newell EW, Ramey J, Davis MM et al. OpenCyto: an open source infrastructure for scalable, robust, reproducible, and automated, end-to-end flow cytometry data analysis. PLoS Comput Biol. 2014; 10(8):e1003806.	version 2.2.0
flowStats	Hahne F, Khodabakhshi AH, Bashashati A, Wong CJ, Gascoyne RD, Weng AP et al. Per-channel basis normalization methods for flow cytometry data. Cytometry A. 2010; 77(2):121-31.	version 3.40.1
FlowSOM	Nowicka M, Krieg C, Crowell HL, Weber LM, Hartmann FJ, Guglietta S et al. CyTOF workflow: differential discovery in high-throughput high-dimensional cytometry datasets. F1000Res. 2017; 6:748.	version 1.14.1
GLIPH2	Chiou SH, Tseng D, Reuben A, Mallajosyula V, Molina IS, Conley S et al. Global analysis of shared T cell specificities in human non-small cell lung cancer enables HLA inference and antigen discovery. Immunity. 2021; 54(3):586–602 e8.	
Immunarch	https://immunarch.com/	

### Resource availability

#### Lead contact

For additional details and inquiries regarding resources and reagents, kindly reach out to Alessandra Petrelli at petrelli.alessandra@hsr.it, who will handle your requests.

#### Materials availability

This study did not generate new unique reagents.

#### Data and code availability

Data reported in this paper will be shared by the lead contact upon request. Raw and processed data of RNA-seq and TCR-seq are deposited in Gene Expression Omnibus (accession number GSE237338). Any additional information required to reanalyse the data reported in this paper is available from the lead contact upon request.

### Experimental model and study participant details

Obese patients undergoing bariatric surgery were enrolled at Ospedale San Raffaele (OSR), Milan and Policlinico S. Marco, Zingonia (BG), Italy from February 2017 to June 2022. Inclusion criteria were age 18–65 years and body mass index (BMI) ≥35 kg/m^2^. Exclusion criteria were presence of acute or chronic infections, concomitant autoimmune or chronic inflammatory diseases, including malignancies, and severe cardiovascular disease. Obese patients were stratified in two groups based on their diabetes status, i.e., patients with normoglycemia (OB-ND) and patients with dysglycemia (OB-Dys), according to the American Diabetes Association (ADA) guidelines.^51^ OB-Dys group included both patients with type 2 diabetes (OB-D) and with prediabetes (OB-PreD). Patients with fasting glucose <100 mg/dL and/or hemoglobin A1c (HbA1c) < 5.7% were classified as OB-ND. Patients with fasting glucose ≥126 mg/dL and/or hemoglobin A1c (HbA1c) ≥6.5% or undergoing treatment with antidiabetic medications were classified as OB-D. Patients with fasting glucose ≥100 and ≤125 mg/dL or with HbA1c 5.7–6.4% were classified as OB-PreD. Diabetes status was assessed based on blood tests available in the last 4 months before surgery. Healthy lean controls (LC) were enrolled for some experiments. They had a BMI ≤25 kg/m^2^ and were selected from the waiting list for living kidney donation at OSR. LC were free from diabetes, concomitant infections, malignancies, autoimmune and chronic inflammatory diseases. Clinical data, including BMI, fat mass, lipid profile, renal function, fasting glycemia, fasting insulin, HbA1c and blood tests were obtained from all patients prior to surgery. The Homeostasis Model Assessment of Insulin Resistance (HOMA-IR) was calculated using the formula: fasting glucose (mg/dL)∗fasting insulin (mU/L)/405, as illustrated in Matthews et al.^52^ The figure legend provides information on the quantity of subjects associated with each experiment. Liver biopsies from n = 12 patients with histological evidence of NASH were studied by the OSR Pathology Unit, 7/12 individuals were dysglycemic, while 5/12 were normoglycemic. The Bedossa score was available for 10 out of 12 patients and was 4 in 6/12, 6 in 2/12 and 7 in 2/12. BMI, available for 5/12 subjects, was in the overweight range. The study was approved by the OSR Ethics Committee. All the study participants signed the informed consent (protocols DRI006-FAT001 and DRI-FAT003).

### Method details

#### Sample collection and processing

Visceral adipose tissue (VAT) was collected from OB-ND, OB-Dys and LC during surgery. In particular, omental fat was collected from obese patients and perirenal fat from LC. Blood was withdrawn before general anesthetic injection. VAT was transferred to the laboratory in Dulbecco’s Modified Eagle Medium (DMEM) supplemented with GlutaMAX (ThermoFisher – Scientific), bovine serum albumin (BSA), penicillin/streptomycin (P/S), and HEPES, where it was processed within 90 min from the collection. VAT was finely minced and digested with collagenase IV (Sigma-Aldrich) resuspended in PBS with a final concentration of 2 mg/mL for 40 min at 37°. The sample was then washed with DMEM high glucose (10% FBS, 1% P/S, 1% Glutamine) and treated with Red Blood Lysis buffer (Biolegend) to obtain the stromal vascular fraction (SVF). After washing, the pellet of SVF was filtered and counted. Whole blood (WB) was collected simultaneously with VAT and used for flow cytometry analysis. Peripheral blood mononuclear cells (PBMCs) were obtained using Lympholyte Cell Separation density gradient centrifugation media (Cedarlane). After centrifugation, the PBMC ring was collected and washed two times with PBS.

#### Flow cytometry analysis

SVF (1×10^6^ cells) and WB were suspended in X-Vivo medium supplemented with human serum and P/S. Stainings were performed within 24 h from tissue collection as described in Cardellini et al.^13^ The list of anti-human monoclonal antibodies, including information on the company catalog number, clone and concentration used for our assays is provided in Table S3. Cells were counted and washed with PBS and stained with Fixable Viability Stain 575V (BD) before antibody incubation. Annexin staining was performed according to manufacturer’s instructions (BD) on samples marked with antibody and live dead staining. Apoptotic cells were identified as Annexin positive within the live-dead negative fraction. Data were acquired on a BD LSRFortessa or FACSymphony instruments (BD Biosciences). Fluorescence intensity was standardized using multiple peak Rainbow Calibration Particles (Spherotech). For unsupervised analysis, T cells were extracted, fcs. files were imported and logicle transformed in R environment (v. 4.0.3). Then, CD4 T cells were isolated using OpenCyto package (version 2.2.0).^53^ All markers satisfying the rules for normalization have been subjected to the landmark alignment procedure, using the normalize() function in the flowStats R package (version 3.40.1).^54^ Unsupervised analysis was realized using the FlowSOM algorithm, included in the CyTOF/CATALYST pipeline (version 1.14.1).^55^ Uniform Manifold Approximation and Projection (UMAP) and visual representations were realized using the CyTOF/CATALYST pipeline. For the supervised analysis of flow cytometry data, FlowJo V.10 was used.

#### Cell sorting

SVF and PBMC samples were thawed, washed with PBS and incubated with Fixable Viability Dye (Invitrogen, 65-0866-14). Then, samples were washed with 10% FBS RPMI medium, incubated with fluorescent conjugated antibodies and sorted using FACSAria Fusion (BD). The following antibodies were used: PD-1 PE (Invitrogen), CD127 PE-Cy7 (Beckman Coulter), CD45 PB (Biolegend), CD8 BV605 (Biolegend), CD25 APC (BD), CD4 BV711 (Biolegend), CD3 PerCP (Biolegend), Additional information are provided in Table S3. Sorted cells were collected in RPMI medium supplemented with 10% FBS (Lonza) and washed with PBS before RNA extraction.

#### RNA extraction

Total RNA was obtained from sorted cells using RNeasy Micro Kit (Qiagen), according to manufacturer procedures. RNA integrity and concentration was determined using the 2200 TapeStation instrument (Agilent Technologies).

#### RNA sequencing and analysis

RNA libraries were prepared using the SMART-Seq v4 Ultra Low Input RNA Kit for Sequencing (Takara Bio USA), according to the manufacturer’s instructions. Sequencing of PD-1+ and PD-1- CD4 Tconv cells sorted from SVF and PBMCs of OB-ND and OB-Dys was performed on Illumina NextSeq 500 and Illumina NovaSeq 6000 platforms (Illumina, San Diego, CA) obtaining an average of 30 millions of paired-end reads per sample. The raw reads produced from sequencing were trimmed using Trimmomatic, version 0.32, to remove adapters and to exclude low-quality reads from the analysis. The remaining reads were then aligned to the human genome hg38 using STAR, version 2.5.3a. Reads were assigned to the corresponding genomic features using featureCounts, according to the Gencode basic annotations (Gencode version 31). Quality of sequencing and alignment was assessed using FastQC, RseQC and MultiQC tools. Expressed genes were defined as those genes showing at least 1 Count Per Million reads (CPM) on at least a selected number of samples, depending on the size of the compared groups. Low-expressed genes that did not match this criterion were excluded from the corresponding dataset. Gene expression read counts were imported and analyzed in R environment (version 4.0.3) to identify differentially expressed genes (DEGs) using the DESeq2 Bioconductor library6 (v. 1.30.1). Significant genes were identified as those genes showing nominal p value < 0.01 and a Log2 Fold Change with an absolute value greater than 1.^56^ Functional enrichment analysis was conducted using the enrichR web-based platform,^57^ starting from the lists of differentially expressed genes. Pre-ranked Gene Set Enrichment Analysis (GSEA)^58^ was performed for each DGE comparison, on all the expressed genes ranked according to their Log2 Fold Change. The tested signatures were retrieved from the Gene Ontologies, Hallmarks, Immunologic signatures and Canonical Pathways collections, as reported in the MSigDB. The Pearson Correlation Coefficient was calculated between Log2RPKM expression values and HOMA-IR coefficients, considering genes with correlation coefficient >0.8 and p value <0.05 as positively correlated.

#### TCRβ sequencing

Sequencing of the TCRβ chain was performed on PD-1^+^ and PD-1^−^CD4 Tconv cells sorted from SVF and PBMCs of OB-ND, OB-Dys and LC. RNA was extracted using a RACE PCR protocol. cDNA was synthesized using the SmartScribe enzyme (Clontech) in the presence of primers specific for the constant β chain genes and of a barcoded (5 bp) template switching primer. Gene-specific cDNA and first PCR product were used as input material for the first and second exponential PCR, respectively. Fusion-primers harboring Illumina MiSeq sequencing adaptors were added in the second exponential PCR. Each individual sample was tagged with a barcoded (10 bp) fusion primer specific for the constant TCR genes. PCR amplicons were purified using the AmPure beads (Beckman Coulter), quality checked on the Tape station (Agilent) and sequenced with the MiSeq reagent kit v3 (paired end sequencing, 2 × 300 bp). Raw data were processed to discard low quality sequences (Phred score >Q30) and demultiplexing of the sequencing results performed according to the unique primer combination used for the barcoding.

#### TCRβ sequencing analysis

Analysis of the TCRβ clonotypes was carried out using the MiXCR software and the VDJ tool to remove non-functional (non-coding) clonotypes and to perform frequency-based correction to eliminate erroneous clonotypes. TCRβ sequences were analyzed using Immunarch R package to calculate diversity indexes and to estimate Gene usage. Identification of antigen-specific T cell clusters was obtained by implementing GLIPH2 algorithm, as described in Chiou et al.^59^ Out of 2884 TCR patterns identified by GLIPH2 algorithm, 241 were retained for analysis after filtering out motifs that did not meet the following criteria: number of unique complementarity determining regions 3 (CDR3s) in the cluster ≥3, Fisher_score ≤0.05, p value for the Vβ gene enrichment in the indicated specificity group ≤0.05, CDR3β length p value for the bias in length distribution in the indicated specificity group ≤0.05. The curated database of TCR sequences VDJdb (https://vdjdb.cdr3.net/)^60^ was exploited to identify antigen specificities of amino acid sequences. TCR amino acid sequences and TCR motifs (or clusters) identified by GLIPH2 algorithm [32] were entered in VDJdb. TCR sequences with a VDJdb score <1 and those associated with CD8 T cells or class I MHC were filtered out.

#### Immunofluorescence on liver biopsies

For double immunofluorescence staining, 3-μm-thick sections paraffin embedded sections underwent antigen retrieval with solution CC1(Ventana) for 56 min, then incubated with primary antibody anti-PD1 (Cell Marque, NAT105, Mouse Monoclonal Antibody) for 16 min, then Ultra map anti-Ms HRP for 12 min and colored DISCOVERY FITC Kit. Then denaturation occurred and sections were incubated with primary anti-CD4 (Ventana, CONFIRM SP35, Rabbit Monoclonal Primary Antibody) for 26 min, then incubated with Ultra map anti Rb- HRP for 12 min and colored with DISCOVERY RED 610 Kit. The protocol was performed with DISCOVERY Ultra Ventana machine. Then the sections were washed and hand-stained with DAPI for the nuclei (Vectashield) and covered. The sections were evaluated under the bright-field and fluorescent microscope (a Nikon Eclipse 90i, Nikon Instruments). For each case one section has been evaluated and the maximum number of cells with colocalization of CD4 and PD1 has been measured.

### Quantification and statistical analysis

All values are reported as median ± interquartile range. Differences between groups were determined using Mann-Whitney U test (2 groups) Kruskal-Wallis test (>2 groups), Wilcoxon matched pairs signed ranked test was adopted for paired measure. Pearson Correlation Coefficient has been used to measure linear correlation. Data were analysis using Graph Pad Prism 7 software or R environment (version 4.0.3). p values less than 0.05 were considered significant. Plots were created using the ggplot2 (Wickham, 2016) and pheatmap2 (version 1.0.12) R packages.

## Supplemental information

Document S1. Figures S1–S13 and Table S1

Table S2. List of genes positively correlated with HOMA-IR (Pearson correlation coefficient >0.8; p-value <0.05), related to Figure 2
