# Supplementary material for: Fat-to-blood recirculation of partially dysfunctional PD-1+CD4 Tconv cells is associated with dysglycemia in human obesity
Source: iScience. 2024 Feb 1;27(3):109032. doi: 10.1016/j.isci.2024.109032 (PMC10877684; doi:10.1016/j.isci.2024.109032)
Supplement: Document S1. Figures S1–S13 and Table S1 [file mmc1.pdf]

iScience, Volume *27*

Supplemental information

Fat-to-blood recirculation of partially dysfunctional PD-1^{+}CD4 Tconv cells is associated with dysglycemia in human obesity

Anna Giovenzana, Eugenia Bezzecchi, Anita Bichisecchi, Sara Cardellini, Francesca Ragogna, Federica Pedica, Federica Invernizzi, Luigi Di Filippo, Valentina Tomajer, Francesca Aleotti, Giulia M. Scotti, Carlo Socci, Giovanni Cesana, Stefano Olmi, Marco J. Morelli, Massimo Falconi, Andrea Giustina, Chiara Bonini, Lorenzo Piemonti, Eliana Ruggiero, and Alessandra Petrelli

SUPPLEMENTAL INFORMATION

Supplementary Tables

	baseMean	log2Fold-Change	lfcSE	stat	pvalue
TNFRSF4	258.2243462	3.329142472	0.829310432	4.014350168	5.96E-05
CCR7	1046.456933	1.87287904	0.503131396	3.722445181	0.000197303
RFX2	247.9036059	3.176346268	0.87792304	3.618023591	0.000296861
BASP1	86.20471459	5.656171553	1.57192855	3.598237052	0.000320382
CD109	163.218857	3.187249983	0.896927929	3.553518492	0.000380114
AP003086.1	388.1865847	5.193902014	1.468554234	3.536745116	0.00040509
RARG	158.2567229	2.678286859	0.776349263	3.449847881	0.000560903
PIK3AP1	160.9756242	1.920429212	0.557854284	3.442528395	0.000576303
SECTM1	99.96782053	3.023610945	0.894387464	3.380649959	0.000723146
ITGAX	573.2603737	4.092067341	1.232515103	3.32009509	0.000899868
S1PR4	408.1099406	1.71514885	0.525794476	3.262013828	0.001106237
CTLA4	692.417213	1.516596225	0.476491096	3.182842738	0.001458368
ZNF704	46.94500085	3.094776617	0.977350556	3.16649599	0.001542875
ANKRD26P4	15.73658942	7.132997908	2.261569938	3.154002796	0.001610475
VDR	134.4786573	2.42325197	0.769027853	3.151058781	0.001626797
SLC7A11	21.68993364	5.894066395	1.888335907	3.121301869	0.001800534
IL1RN	733.7228872	3.896459931	1.251284169	3.113968855	0.00184589
SLCO4A1	256.0081923	2.407423892	0.780543495	3.084291789	0.002040374
CD177	13.16455607	6.881263862	2.235755438	3.077824947	0.002085174
SEC24D	323.7282998	1.745127999	0.567929582	3.072789398	0.002120681
EREG	1170.485036	3.603190262	1.184551426	3.041818349	0.002351538
RUBCNL	40.76182181	3.018939533	1.003149658	3.009460761	0.002617119
CCR8	35.81358111	2.794352801	0.929314121	3.006898031	0.002639283
NEU4	36.12053687	3.596272849	1.210727747	2.970339828	0.002974705
ARHGEF11	55.42400628	4.610010789	1.609042575	2.865064518	0.004169243
SPI1	73.13320788	3.952539623	1.380981375	2.862123772	0.004208125
DYSF	44.80196136	5.099273135	1.789456215	2.849621629	0.004377127
MEFV	24.31527752	5.531255876	1.949410457	2.837399306	0.004548269
SLC22A4	106.2970459	2.849760806	1.007349545	2.82896917	0.00466982
HMOX1	465.4798758	3.044969249	1.087005835	2.801244621	0.005090591
SEC31B	419.5347016	1.84606864	0.659363832	2.799772372	0.005113865
SLC24A4	33.95914814	4.361393722	1.566246529	2.784615092	0.00535913
PPP4C	271.0953107	1.91597724	0.688649345	2.782224732	0.005398764
TIMP1	2461.358342	3.12730905	1.132252454	2.762024528	0.005744416
PELI1	1374.261211	1.537141193	0.558383949	2.752839146	0.005908092
AC023051.1	15.83670439	3.646960795	1.327462705	2.747316954	0.006008504
VEGFA	145.5768623	3.859337127	1.405028543	2.746803364	0.00601792
GNA15	445.4302691	1.654257624	0.602941679	2.743644503	0.00607613

OCRL	65.5453221	4.223673249	1.545311265	2.733218444	0.006271872
B4GALT5	160.0901734	1.913064453	0.700543953	2.73082716	0.006317559
ZFYVE16	224.130867	1.401610315	0.515207406	2.720477809	0.006518765
PPP4R2	397.5907149	1.411091579	0.519601192	2.715720443	0.006613173
AC092145.1	75.05896901	2.268211983	0.835410582	2.715086486	0.006625846
AC015912.3	125.7729902	3.370209798	1.245249948	2.706452469	0.006800632
AC004890.3	14.46423677	5.787683696	2.146841994	2.69590576	0.007019752
MYO16	68.06836864	4.010822102	1.496038558	2.680961719	0.007341091
PAK3	39.42140505	2.421632358	0.913649193	2.650505661	0.008037138
SCML1	231.0567726	2.07494961	0.786166539	2.639325777	0.008307111
AC027237.1	74.79057638	2.741815333	1.040189476	2.635880669	0.008391924
AC087893.1	17.41337387	3.189016534	1.211746764	2.63175164	0.008494593
STK3	152.7285704	1.927724694	0.734010382	2.626290773	0.008632104
AGPAT4	374.1695654	1.450094675	0.5535456	2.619648091	0.008802055
ALKBH1	74.25952262	2.01522423	0.769807054	2.617830299	0.00884908
GK	311.0658745	2.973338985	1.139173968	2.610083332	0.009052017
TDRD9	31.99366244	4.428007023	1.700122992	2.604521581	0.009200263
FDXACB1	44.23842966	2.915018222	1.122973574	2.595803045	0.009437014
RIPK4	34.10033048	4.233218306	1.639001049	2.582803902	0.009800101
IRAK2	210.6920073	1.57291431	0.609142865	2.582176367	0.00981794

Table S1. List of differentially expressed genes (DEGs) between PD-1+ CD4 Tconv cells of OB-Dys (n= 4) and OB-ND (n= 3).

Antigen/Target	Fluorophore	Company	Clone	Catalogue number	Concentration
CCR7	PE	Biolegend	G043H7	353204	0.67 : 100
CD127	PE-Cy7	Beckman Coulter	R34.34	A64618	2 : 100
CD137	BV786	BD	4B4-1	741000	0.5 : 100
CD25	APC	BD	2A3	340907	2 : 100
CD25	APC-Cy7	Biolegend	M-A251	356122	1.33 : 100
CD3	BUV395	BD	SK7	564001	2 : 100
CD3	PerCP	Biolegend	SK7	344814	0.5 : 100
CD4	BUV805	BD	SK3	612887	1 : 100
CD4	BV711	Biolegend	OKT4	317440	0.33 : 100
CD45	BV510	Biolegend	HI30	304036	0.67 : 100
CD45	PB	Biolegend	HI30	304022	2 : 100
CD45	PerCP-Cy5	Biolegend	HI30	304028	2 : 100
CD45RA	BV421	Biolegend	HI100	304130	1.33 : 100
CD69	BV650	Biolegend	FN50	310934	0.33 : 100
CD8	APC-H7	BD	SK1	560179	1 : 100
CD8	BV605	Biolegend	SK1	344742	0.33 : 100

FoxP3	Alexa Fluor 488	Biolegend	259D	320212	4 : 100
GzmB	Alexa Fluor 647	Biolegend	GB11	515406	1 : 100
HLA-DR	BUV661	BD	G46-6	565074	1 : 100
IFNγ	PB	Biolegend	4S.B3	502522	2 : 100
Ki67	PE eFluor 610	eBioscience	20Raj1	61-5699-42	0.5 : 100
LAG-3	PE-CF594	BD	T47-530	565719	1 : 100
Live/dead	575V	BD		565694	1 : 1000
Live/dead	eFluor506	Invitrogen		65-0866-14	1 : 1000
PD-1	R718	BD	EH12.1	566974	1 : 100
PD-1	PE	Invitrogen	eBioJ105	12-2799-42	2 : 100
PD-1	PE-Cy7	eBioscience	eBioJ105	25-2799-42	1.33 : 100
TIGIT	BV711	BD	741182	747839	0.5 : 100
TIM-3	BUV737	BD	7D3	748820	0.5 : 100
TNFSRF4	PE-Cy5	BD	ACT35	551500	2 : 100
TNFα	BV650	Biolegend	MAb11	502938	0.5 : 100
CD57	PB	Biolegend	HNK-1	359608	2 : 100

Table S3. List of markers used for flow cytometry immunophenotyping.

Supplementary Figures

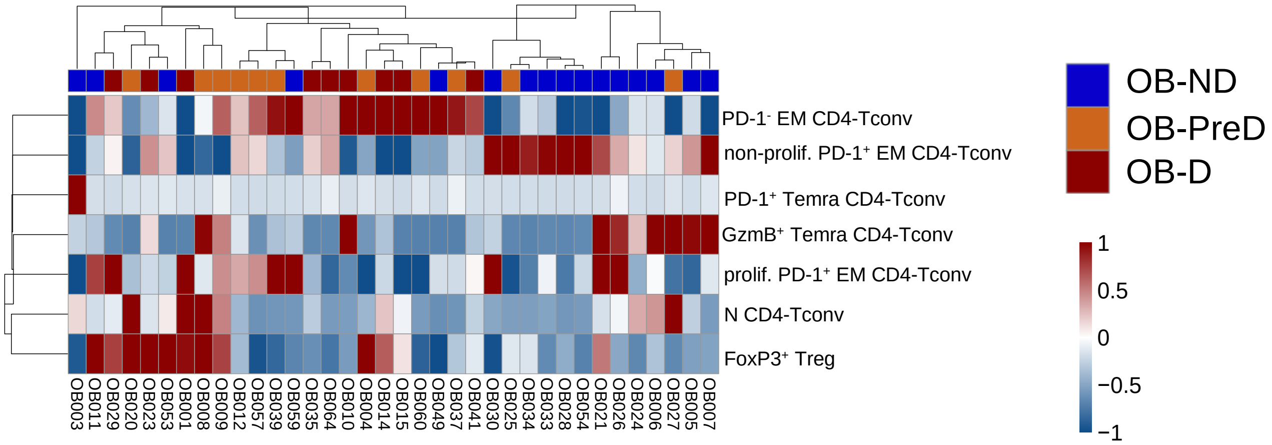

Figure S1. Heatmap of CD4 T cell clusters in the VAT of obese patients, related to Figure 1. Heatmap showing color-coded expression of CD4 T cell clusters identified by FlowSOM algorithm. The dendrograms indicate hierarchical clustering of OB patients. *OB-ND: obese patients with normoglycemia (n= 16); OB-PreD: obese patients with prediabetes (n= 11); OB-D: obese patients with type 2 diabetes (n= 9).*

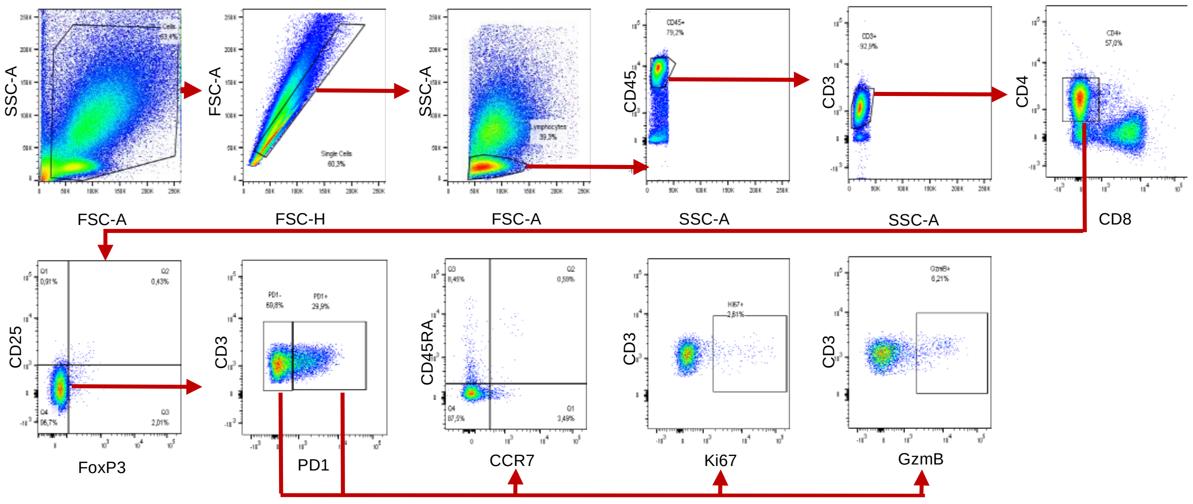

Figure S2. Gating strategy used for supervised analysis of flow cytometry data, related to Figure 1.

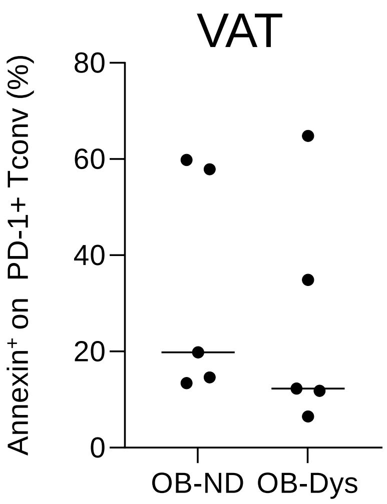

Figure S3. Frequency of Annexin+ cells on PD-1^{+} CD4 Tconv from the VAT obese patients with and without dysglycemia, related to Figure 1. Frequency of Annexin+ cells on live (cells negative for live-dead staining) PD-1+ CD4 Tconv cells in VAT of OB-Dys and OB-ND (n=5 per group). Data are presented as median. Statistical analysis: Mann Whitney test. *OB-ND: obese patients with normoglycemia; OB-Dys: obese patients with dysglycemia.*

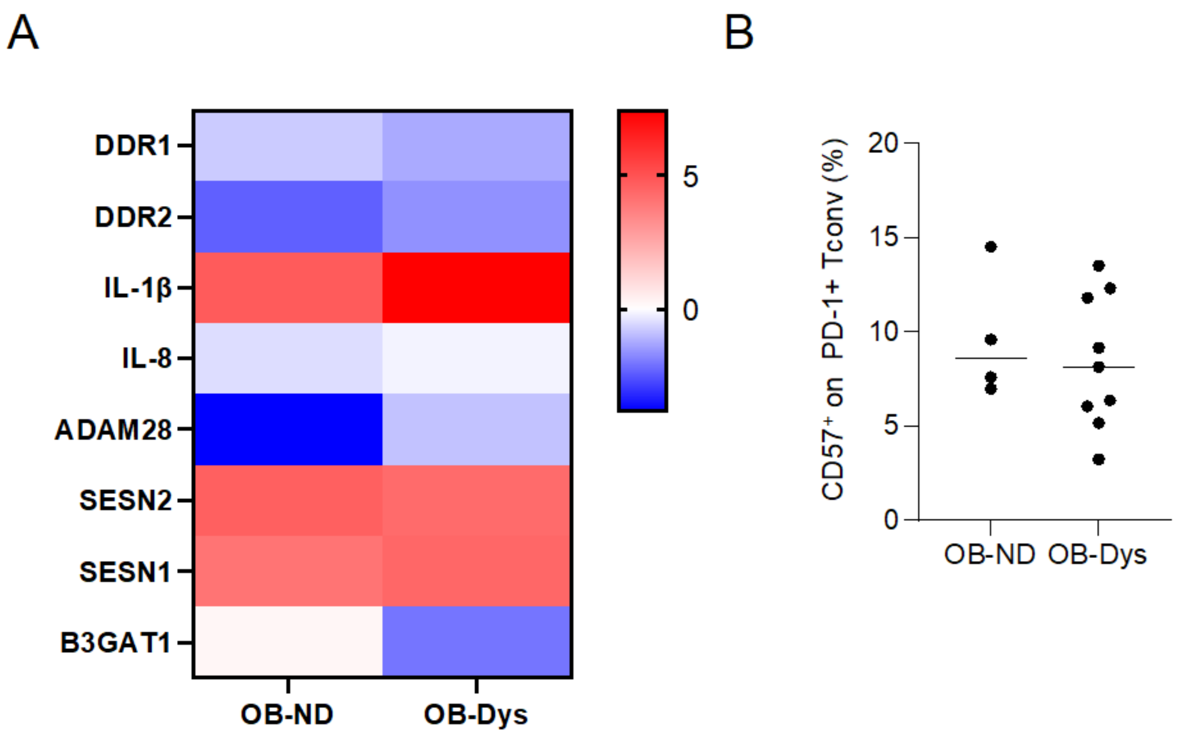

Figure S4. Dysglycemia is not associated with senescence of VAT-derived PD-1$^+$ CD4 Tconv cells, related to Figure 2. (A) Heatmap showing the expression of senescence-associated markers on RNA-sequencing data of PD-1$^+$ CD4 Tconv cells from OB-Dys (n=4) and OB-ND (n=3). (B) Median percentage of CD57 in PD-1$^+$ CD4 Tconv cells of OB-Dys (n=4) and OB-ND (n=9). Data are presented as the median. Statistical analysis: Mann Whitney test. *OB-ND: obese patients with normoglycemia; OB-Dys: obese patients with dysglycemia.*

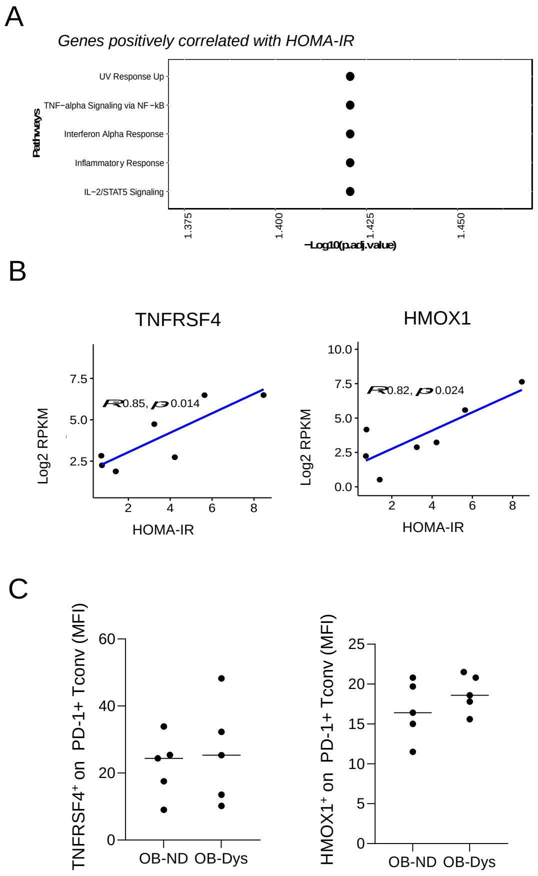

Figure S5. Genes positively correlated with HOMA-IR levels are enriched in pathways associated with inflammatory responses, related to Figure 2. Two-hundred twenty-seven genes were found to be positively correlated with HOMA-IR levels in PD-1^{+} CD4 Tconv cells. (A) Pathway enrichment analysis. (B) Scatter plots of *TNFRSF4* and *HMOX1*. showing correlation between gene expression levels and HOMA-IR. Pearson correlation coefficient (R) and p-values (p) are shown in the graphs. OB-ND (n=3). OB-Dys (n=4). (C) Median fluorescence intensity of TNFRSF4 (left) and HMOX1 (right) on PD-1^{+} CD4 Tconv cells in the VAT of obese patients (n=5 per group). Data are presented as the median. Statistical analysis: Mann Whitney test. *OB-ND: obese patients with normoglycemia; OB-Dys: obese patients with dysglycemia.*

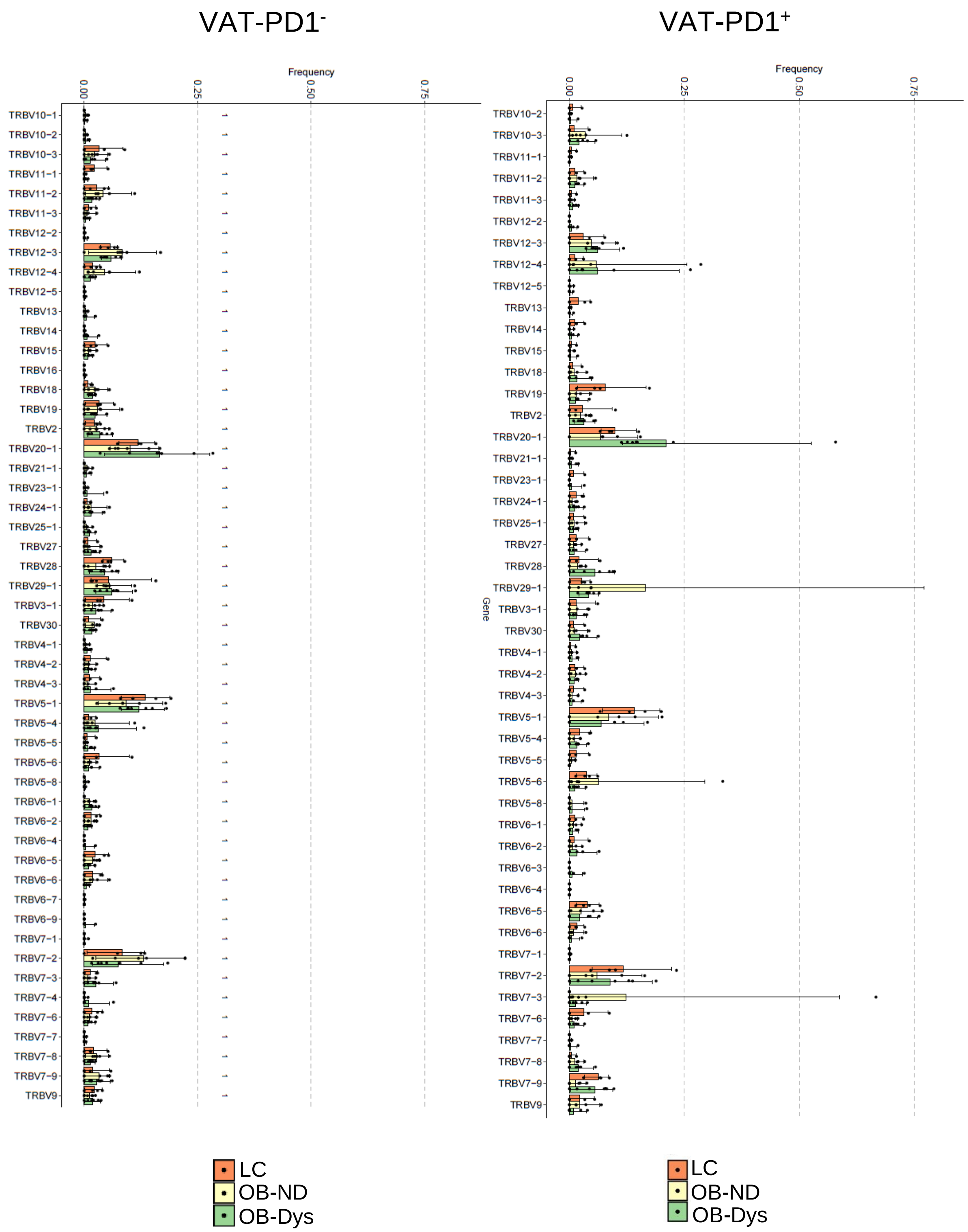

Figure S6. Frequency of TCRVβ gene families in PD-1$^+$ and PD-1$^-$ CD4Tconv from the VAT of obese patients and lean controls, related to Figure 3. *LC: lean non-diabetic control; OB-ND: obese patients with normoglycemia; OB-Dys: obese patients with dysglycemia.* LC (n= 4). OB-ND (n= 6) and OB-Dys (n= 7)

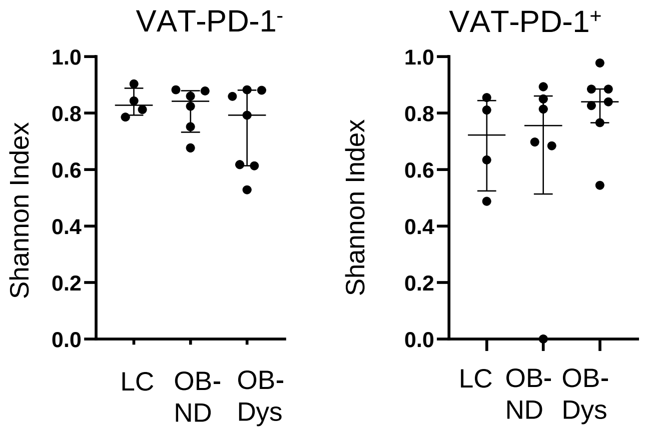

Figure S7. Diversity of the TCRβ repertoire of VAT-derived PD-1$^+$ and PD-1$^-$ CD4 T conv cells, related to Figure 3. Graphs indicate the median value of Shannon Index for VAT-derived PD-1$^+$ (A) and PD-1$^-$ (B) CD4 T conv cells from LC (n=4). OB-ND (n=6) and OB-Dys (n=7) individuals). Data are presented as the median + IQR. *LC: lean non-diabetic control; OB-ND: obese patients with normoglycemia; OB-Dys: obese patients with dysglycemia.*

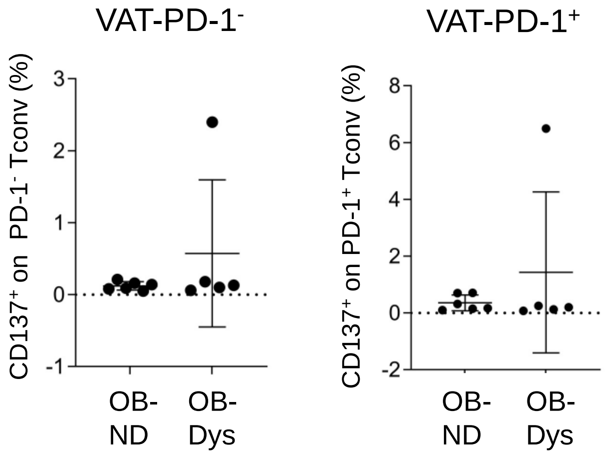

Figure S8. Frequency of CD137$^+$ cells on CD4 Tconv in the VAT of obese patients, related to Figure 3. Median frequency of CD137$^+$ cells on PD-1$^-$ and PD-1$^+$ CD4 Tconv cells in the VAT of obese patients (n=5 per group). Data are presented as the median + IQR. Statistical analysis: Mann Whitney test. *OB-ND: obese patients with normoglycemia; OB-Dys: obese patients with dysglycemia.*

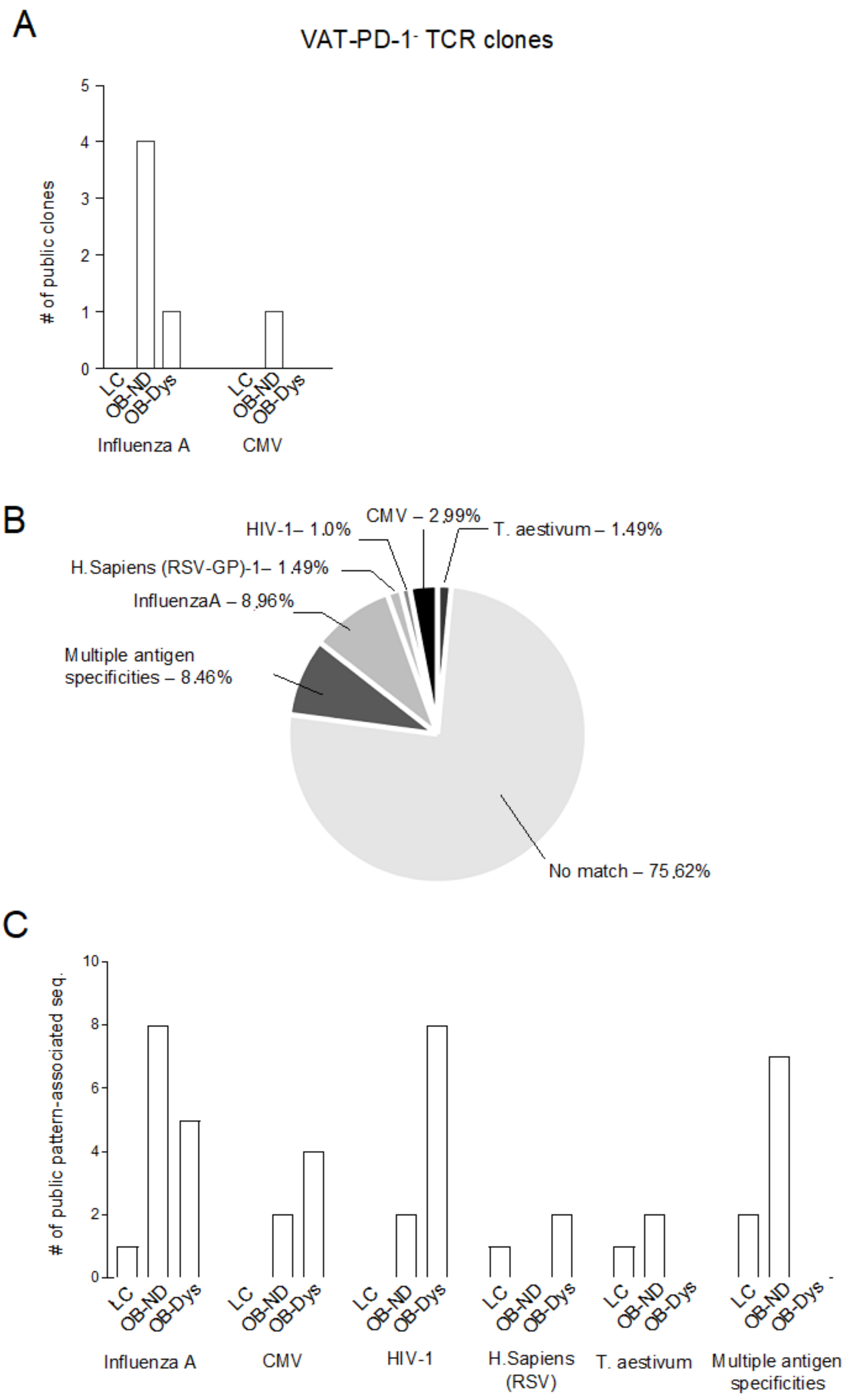

Figure S9. Antigen-specificity of the TCRβ repertoire of VAT-derived PD-1^{-}CD4 Tconv cells, related to Figure 3. A) Number of clones with a unique match in VDJdb. B) Pie chart showing the frequency of patterns with a match for antigens annotated in VDJdb. C) Number of pattern-associated sequences with a unique match in VDJdb. In “multiple antigen specificities" bars indicate the number of pattern-associated sequences matching with multiple antigens. LC (n= 4). OB-ND (n= 6) and OB-Dys (n= 7).

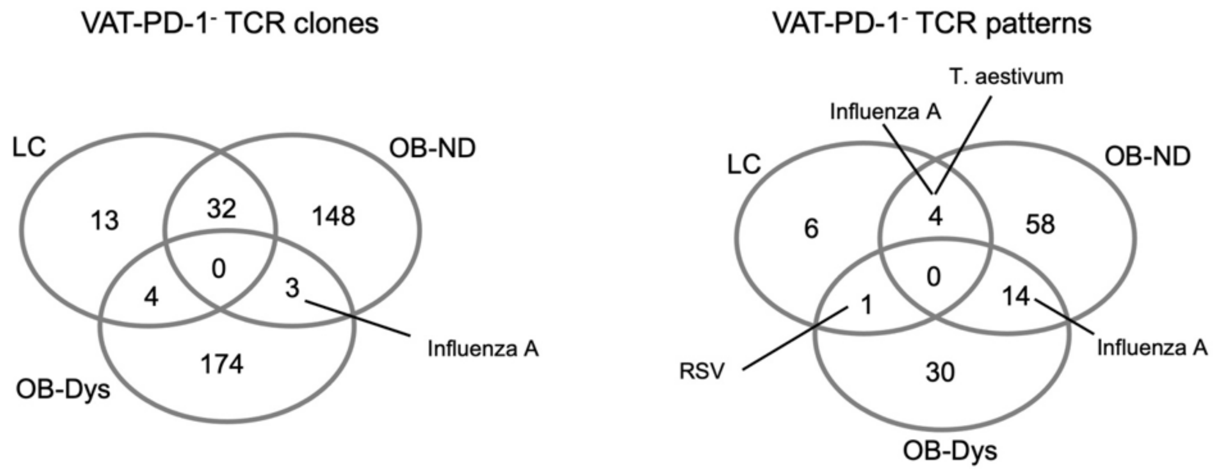

	P-value	Odds Ratio
LC - OB-ND	0.0146	2.169
LC - OB-Dys	<0.0001	0.074
OB-ND - OB-Dys	<0.0001	0.001

	P-value	Odds Ratio
LC - OB-ND	0.08	0.24
LC - OB-Dys	0.05	0.13
OB-ND - OB-Dys	0.0001	0.04

Figure S10. Heterogeneity of the TCRβ repertoire of VAT-derived PD-1$^-$CD4 Tconv cells, related to Figure 3. A) Venn diagrams showing the number of clones (left panel) and patterns (right panel) shared among LC. OB-ND and OB-Dys individuals in VAT-derived PD-1$^-$CD4 Tconv cells. Matches with public clones annotated in VDJdb are reported. Exact Fisher's test has been adopted to evaluate the association between shared and unshared clones and patterns for each comparison (LC vs OB-ND, LC vs OB-Dys, OB-ND vs OB-Dys). LC (n= 4), OB-ND (n= 6) and OB-Dys (n= 7).

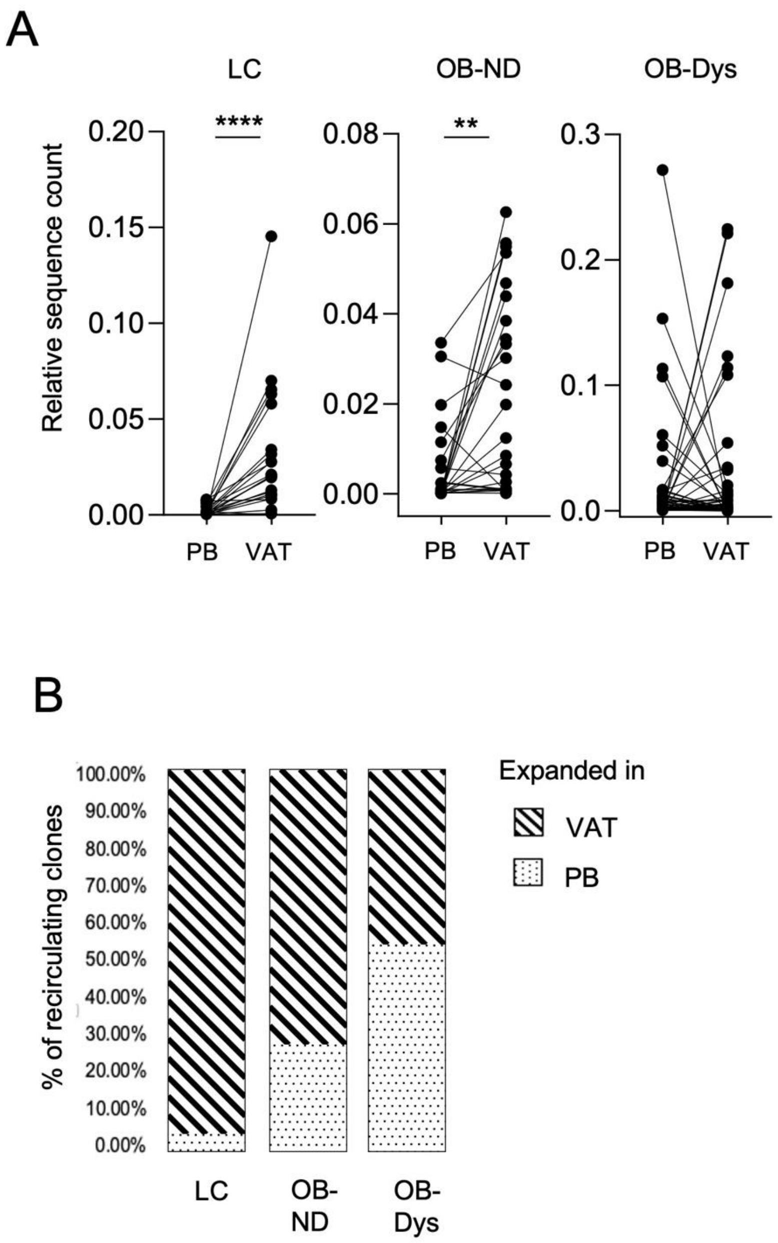

Figure S11. Recirculating PD-1^{-}CD4 Tconv cell clones in OB-Dys are more expanded in PB, related to Figure 4. A) Relative sequence count of recirculating PD1-CD4 Tconv cells in PB and VAT in LC. OB-ND and OB-Dys. Statistical analysis: Wilcoxon test. B) Stacked bar plots showing the percentage of recirculating clones of LC. OB-ND and OB-Dys whose relative sequence count is higher in VAT (stripes) or in PB (dots). LC (n= 4). OB-ND (n= 6) and OB-Dys (n= 7). $*p < 0.05$, $**p < 0.01$, $***p < 0.001$, $****p < 0.0001$.

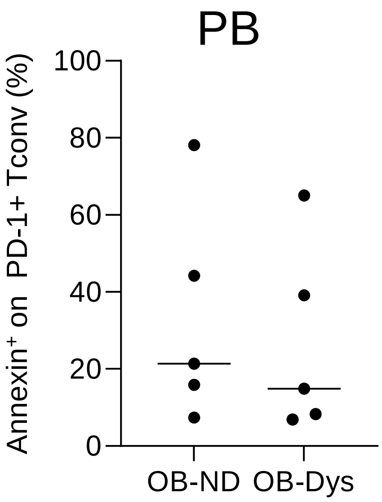

Figure S12. Frequency of Annexin+ cells on PD-1+ CD4 Tconv from the PB obese patients with and without dysglycemia, related to Figure 4. Frequency of Annexin+ cells on live (cells negative for live-dead staining) PD-1+ CD4 Tconv cells in PB of OB-Dys and OB-ND (n=5 per group). Data are presented as the median + IQR. Statistical analysis: Mann-Whitney test. *OB-ND: obese patients with normoglycemia; OB-Dys: obese patients with dysglycemia.*

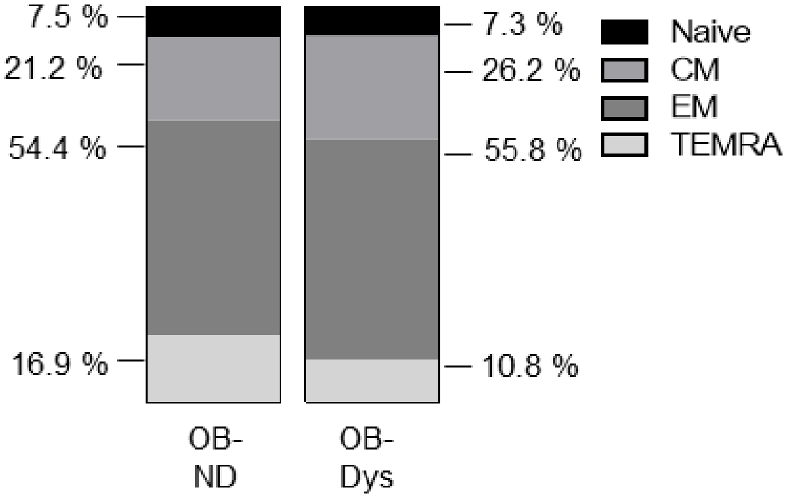

Figure S13. Frequency of naïve and memory phenotypes on PD-1⁺ CD4 Tconv from the PB obese patients, related to Figure 4. Median frequency of naive (CCR7+CD45RA+), central memory (CM, CCR7+CD45RA-), effector memory (EM, CCR7-CD45RA-) and terminally differentiated effector memory cells (TEMRA, CCR7-CD45RA+) on PD-1+CD4 Tconv cells from PB of OB-ND and OB-Dys. Statistical analysis: Mann-Whitney U test. *OB-ND: obese patients with normoglycemia (n = 18); OB-Dys: obese patients with dysglycemia (n = 19).*
